# Glycogen Storage Disease Type Ia: A Retrospective Claims Analysis of Complications, Resource Utilization, and Cost of Care

**DOI:** 10.36469/001c.125886

**Published:** 2025-01-07

**Authors:** Eliza Kruger, Justin Nedzesky, Nina Thomas, Jeffrey D. Dunn, Andrew A. Grimm

**Affiliations:** 1 Ultragenyx Pharmaceutical Inc., Novato, CA, USA; 2 Cooperative Benefits Group, Midvale, UT, USA

**Keywords:** glycogen storage disease type Ia, complications, hospitalization, resource utilization, healthcare costs

## Abstract

**Background:** Glycogen storage disease type Ia (GSDIa) is a rare inherited disorder resulting in potentially life-threatening hypoglycemia, metabolic abnormalities, and complications often requiring hospitalization. **Objective:** This retrospective database analysis assessed the complications, resource utilization, and costs in a large cohort of patients with GSDIa. **Methods:** We conducted a retrospective cohort study of GSDIa patients and matched non-GSDIa comparators utilizing the PharMetrics® Plus database. International Classification of Diseases, Tenth Revision (ICD-10) diagnosis codes in any billing position for inpatient and outpatient claims (January 2016–February 2020) were identified for complications related to GSDIa. Healthcare use and costs were assessed by setting of care (inpatient, outpatient, physician office, emergency department, and pharmacy). **Results:** Overall, 557 patients with GSDIa and 5570 matched comparators (male, 63%; adults, 67%) were identified. The most frequent complications in patients with GSDIa vs comparators included anemia due to enzyme disorders (odds ratio, 4.0 × 103; 95% confidence interval, 555.9–2.8 × 104), hepatocellular adenoma (305.9; 41.6–2.2 × 104), liver transplantation (164.6; 21.8-1.2 × 103), and gastrostomy (152.2; 61.1-379.2), as well as acidosis (45.5; 29.4-70.3), hepatomegaly (43.6; 29.1-65.3), hyperuricemia (23.6; 11.9-46.9), and hypoglycemia (20.2; 14.3-28.7). Chronic complications (eg, gout, osteoarthritis, chronic kidney disease, and neoplasms) were more common in adults with GSDIa, whereas acute complications (eg, poor growth, gastrostomy, seizure, and hypoglycemia) were more common in children with GSDIa. Patients with GSDIa more often required hospitalization (0.53 vs 0.06 hospitalizations per patient per year) vs comparators, including 2 or more hospitalizations (26.6% vs 2.3%), longer length of stay (3.1 vs 0.4 days), and more annual visits in all care settings, including 4.3 times more visits in the emergency department. Mean annual total healthcare costs were higher for GSDIa patients vs comparators (33 910vs4410). **Discussion:** In this large, retrospective database analysis, complications observed among patients with GSDIa were consistent with prior reports and demonstrate the chronic and progressive nature of the disease. Resource utilization was substantial in GSDIa patients, and mean annual total healthcare costs were almost 8 times higher than those of comparators. **Conclusions:** GSDIa is associated with numerous potentially serious and sometimes fatal complications, extensive resource utilization, and high management costs.

## BACKGROUND

Glycogen storage disease type Ia (GSDIa) is a rare, inherited, autosomal recessive deficiency of glucose-6-phosphatase-α that results in impaired glycogenolysis and gluconeogenesis.^1,2^ The incidence of GSDIa is approximately 1 in 100 000 live births worldwide.^3,4^ Symptoms of GSDIa during infancy and childhood include severe hypoglycemia, hepatomegaly and nephromegaly due to glycogen accumulation, abdominal protuberance, abnormal or delayed growth, developmental delay, short stature, lethargy, and “doll-like” facial features.^3,5–7^ Chronic complications include osteoporosis, chronic renal disease, hepatocellular adenoma, and hepatocellular carcinoma.^3,5–7^

Management of GSDIa is centered on patient monitoring and prevention of hypoglycemia through dietary supplementation with complex carbohydrates, such as uncooked cornstarch (UCCS), which has been used for several decades.^8^ Dietary supplementation has enabled patients with GSDIa to survive into adulthood.^9–11^ However, despite best management practices with UCCS supplementation, chronic complications (eg, such as hepatocellular adenoma, hepatocellular carcinoma, renal disease, and anemia) persist, requiring lifelong care and longer hospital stays, and patients with GSDIa have higher than average mortality.^4,12–18^ Furthermore, maintaining optimal control of blood glucose with UCCS can be challenging due to the need for frequent dosing, particularly among infants and toddlers, as well as the potential for gastrointestinal complications and nutritional deficiencies and the inability to address the underlying cause of the disease.^8,11,12,19–23^

GSDIa is a rare, chronic condition that requires complex, lifelong management due to the risk of serious complications. While the clinical manifestations and challenges in managing GSDIa have been well documented, there has been a notable gap in systematically quantifying the economic burden associated with its complications and the corresponding healthcare resource utilization. To date, no large-scale analyses have comprehensively captured the real-world healthcare costs and resource use linked to GSDIa, making it difficult for healthcare providers and policymakers to fully understand the financial and logistical implications of managing the disease. This study aimed to address this gap by leveraging the IQVIA PharMetrics® Plus database to provide the first large, retrospective assessment of the burden of GSDIa, including disease complications, healthcare utilization, and treatment costs, in a real-world setting.

## METHODS

### Data Source

The IQVIA PharMetrics® Plus is a longitudinal database of adjudicated medical and pharmacy claims. The de-identified integrated data include all paid medical and pharmacy (retail and mail order) claims for more than 150 million members from more than 70 US commercial health plans. The database contains inpatient and outpatient claims, diagnoses, and procedures based on *International Classification of Diseases, Tenth Revision, Clinical Modification* (ICD-10-CM), Healthcare Common Procedure Coding System (HCPCS), and Current Procedural Terminology (CPT) codes. Because the PharMetrics® Plus database is de-identified and compliant with the Health Insurance Portability and Accountability Act, institutional review board approval was not needed.

### Patient Selection

**Figure 1.**
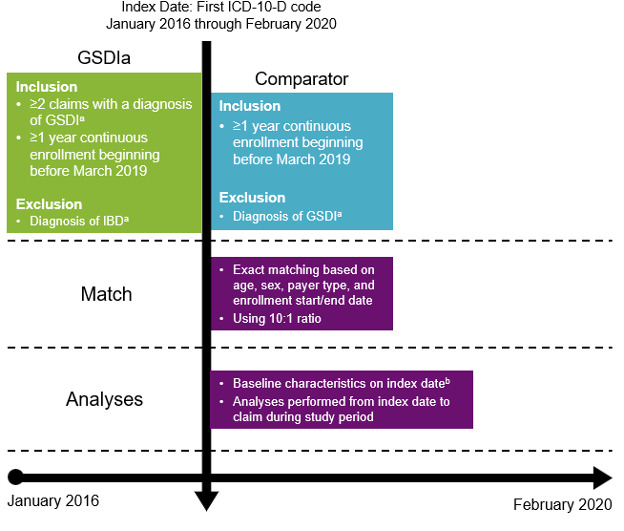
Study Design Abbreviations: GSDIa, glycogen storage disease type Ia; IBD, inflammatory bowel disease; ICD-10, *International Classification of Diseases, Tenth Revision.* ^a^*International Classification of Diseases, Tenth Revision*. Inpatient or outpatient care setting, any diagnosis position. ^b^Baseline characteristics included age, sex, region, and insurance plan type.

PharMetrics® Plus was queried for patients with at least 1 claim and at least 12 months of continuous health plan enrollment during the study period of January 2016 through February 2020. Enrollment was required to begin before March 2019 to ensure at least 1 year of follow-up before impact by the COVID-19 pandemic. For each eligible patient, the first claim during the study period was selected as the index date. Patients were followed up from index to end of continuous enrollment or end of the study period, whichever occurred first. Patients with GSDIa had 2 or more claims with diagnosis of GSDI (E74.01) and no diagnoses related to inflammatory bowel disease (K50.x, K51.x), which may be indicative of GSDIb (**Figure 1**).^3^ A cohort of non-GSDIa comparators was identified using 10:1 exact matching of age, sex, payer type, and continuous enrollment start/end date.^24,25^ A 10:1 ratio (comparator to GSDIa) was selected to ensure adequate sample size in the comparator cohort to capture complications that are rare in a non-GSDIa population and occur more frequently in patients with GSDIa.

### Outcomes

Patient characteristics, including age, sex, geographic region, type of insurance plan, and year were recorded at index. We selected complications of GSDIa for assessment in the GSDIa and comparator cohorts through a literature review and discussions with clinical experts, and we defined them by grouping related ICD-10 diagnosis codes. We identified specified complication codes through inpatient and outpatient medical claims, which could occur in any billing position (**Supplemental Table S1**). We assessed prevalence across all ages and among pediatric and adult groups.

Overall healthcare use and attributable costs were stratified by setting of care: inpatient, outpatient (including emergency department, outpatient hospital, physician office, and other outpatient), and pharmacy. Outpatient visits were defined as unique calendar days where a patient generated a claim. Therefore, more than 1 prescription may have been filled at each pharmacy visit. Event rates and costs were annualized based on mean follow-up time. Costs were adjusted to 2022 US dollars using the medical care component of the Consumer Price Index for urban consumers.^26^

### Statistical Analysis

We summarized study outcomes using descriptive statistics. For categorical variables, we calculated and reported the number and percentage of patients. Continuous variables were presented as mean and SD. To maintain patient confidentiality, we did not report patient counts between 1 and 10. We calculated 95% confidence intervals (CI) and odds ratios (OR) to compare the frequency of complications between the GSDIa and comparator cohorts. For conservative reporting, when counts in the comparator cohort ranged between 1 and 10, we rounded the number up to 10 for OR calculation. In cases where no patients were present in either cohort or where both cohorts had 1 to 10 patients, we did not calculate OR.

All statistical analyses were conducted using SAS Version 9.4, and statistical significance was determined at a threshold of *P* < .05.

## RESULTS

### Study Population

Using the PharMetrics® Plus database, 557 patients with GSDIa and 5570 matched comparators were identified. After matching, demographics between cohorts were balanced. Across both cohorts, 67% were adults, 63% were male, and most were from the South (37%-40%) or Northeast (17%-33%) regions (**Table 1**). Children were 77% male. Most were self-insured (44%-48%) or had commercial insurance (45%-49%).

**Table 1.** Demographic Characteristics

	**Pediatric**		**Adult**		**Total**	
	**GSDIa (n = 185)**	**Comparator (n = 1850)**	**GSDIa (n = 372)**	**Comparator (n = 3720)**	**GSDIa (n = 557)**	**Comparator (n = 5570)**
Mean (SD) age, years	7.4 (5.5)	7.4 (5.5)	41.1 (15.2)	41.1 (15.2)	29.9 (20.4)	29.9 (20.4)
Children, n (%)	185 (100)	1850 (100)	0	0	185 (33.2)	1850 (33.2)
Adults, n (%)	0	0	372 (100)	3720 (100)	372 (66.8)	3720 (66.8)
Sex, n (%)						
Female	42 (22.7)	420 (22.7)	163 (43.8)	1630 (43.8)	205 (36.8)	2050 (36.8)
Male	143 (77.3)	1430 (77.3)	209 (56.2)	2090 (56.2)	352 (63.2)	3520 (63.2)
Geographic region, n (%)						
South	56 (30.3)	699 (37.8)	150 (40.3)	1522 (40.9)	206 (37.0)	2221 (39.9)
Midwest	36 (19.5)	517 (28.0)	76 (20.4)	933 (25.1)	112 (20.1)	1450 (26.0)
Northeast	73 (39.5)	321 (17.4)	109 (29.3)	631 (17.0)	182 (32.7)	952 (17.1)
West	19 (10.3)	295 (16.0)	36 (9.7)	617 (16.6)	55 (9.9)	912 (16.4)
Unknown	1 (0.5)	18 (1.0)	1 (0.3)	17 (0.5)	2 (0.4)	35 (0.6)
Payer type, n (%)						
Commercial	72 (38.9)	848 (45.8)	176 (47.3)	1897 (51.0)	248 (44.5)	2745 (43.7)
Self-insured	99 (53.5)	862 (46.6)	171 (46.0)	1573 (42.3)	270 (48.5)	2435 (43.7)
Medicaid	12 (6.5)	120 (6.5)	12 (3.2)	120 (3.2)	24 (4.3)	240 (4.3)
Medicare	0	0	13 (3.5)	130 (3.5)	13 (2.3)	130 (2.3)
Unknown	2 (1.1)	20 (1.1)	0	0	2 (0.4)	20 (0.4)
Year of first enrollment, n (%)						
2016	127 (68.7)	1270 (68.7)	275 (73.9)	2750 (73.9)	402 (72.2)	4020 (72.2)
2017	29 (15.7)	290 (15.7)	49 (13.2)	490 (13.2)	78 (14.0)	780 (14.0)
2018	25 (13.5)	250 (13.5)	36 (9.7)	360 (9.7)	61 (11.0)	610 (11.0)
2019	4 (2.2)	40 (2.2)	12 (3.2)	120 (3.2)	16 (2.9)	160 (2.9)

Abbreviation: GSDIa, glycogen storage disease type Ia.

### GSDIa Complications

**Figure 2.**
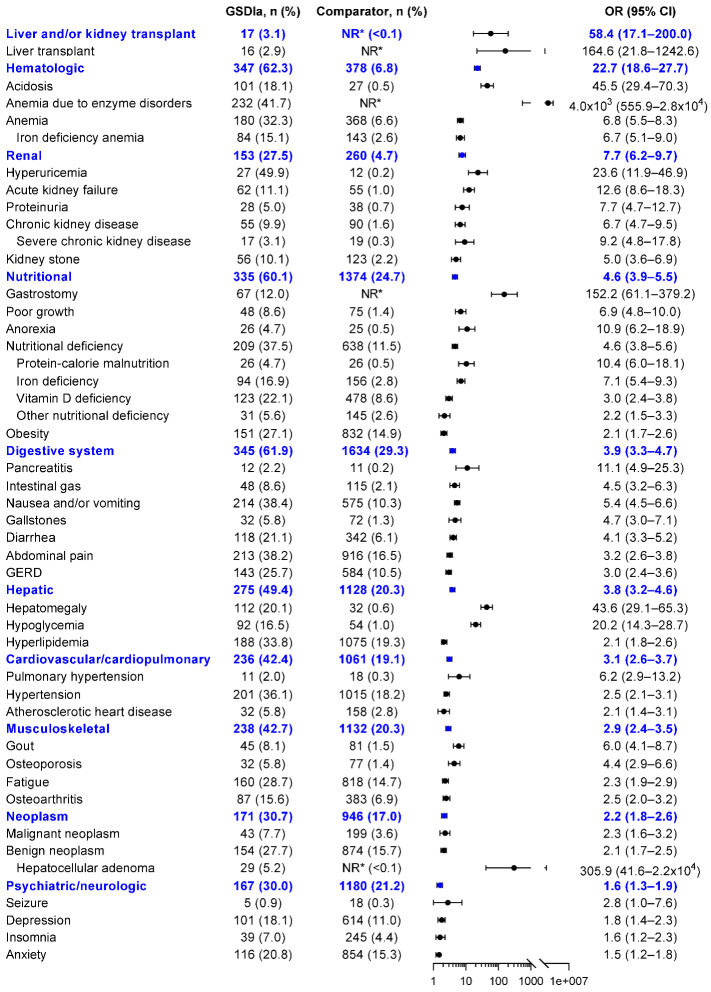
Risk of Complications in Patients With GSDIa vs Comparators Abbreviations: CI, confidence interval; GERD, gastroesophageal reflux disease; GSDIa, glycogen storage disease type Ia; NR, not reported; OR, odds ratio. *Patient counts of 1 to 10 are not reported for privacy reasons.

Among patients with GSDIa, a variety of complications were identified in both inpatient and outpatient settings (**Supplemental Table S2**). The categories of complications that were most likely to occur among patients of any age with GSDIa vs comparators were liver and/or kidney transplantation (OR, 58.4; 95% CI, 17.1-200.0), hematologic (22.7; 18.6-27.7), renal (7.7; 6.2-9.7), nutritional (4.6; 3.9-5.5); and digestive system (3.9; 3.3-4.7) (**Figure 2**). The most frequent complications in patients with GSDIa vs comparators were typically serious or chronic and included anemia due to enzyme disorders (OR, 4.0×10^3^; 95% CI, 555.9–2.8 × 10^4^), hepatocellular adenoma (305.9; 41.6–2.2 × 10^4^), liver transplantation (164.6; 21.8–1.2 × 10^3^), gastrostomy (152.2; 61.1-379.2), acidosis (45.5; 29.4-70.3), hepatomegaly (43.6; 29.1-65.3), hyperuricemia (23.6; 11.9-46.9), hypoglycemia (20.2; 14.3-28.7), acute kidney failure (12.6; 8.6-18.3), and pancreatitis (11.1; 4.9-25.3) (**Figure 2**). Other serious or chronic complications in patients with GSDIa vs comparators included renal disease (7.7; 6.2-9.7) and its other associated complications, such as proteinuria (7.7; 4.7-18.3), chronic kidney disease (6.7; 4.7-9.5), severe chronic kidney disease (9.2; 4.8-17.8), and kidney stones (5.0; 3.6-6.9). Other potentially serious or chronic complications were anorexia (10.9; 6.2-18.9), protein-calorie malnutrition (10.4; 6.0-18.1), anemia (6.8; 5.5-8.3), pulmonary hypertension (6.2; 2.9-13.2), gout (6.0; 4.1-8.7), osteoporosis (4.4; 2.9-6.6), and malignant neoplasm (2.3; 1.6-3.2).

In children with GSDIa vs comparators, the most frequently diagnosed complications were gastrostomy (OR, 195.2; 95% CI, 69.7-546.9), hepatomegaly (185.3; 66.0-519.7), acidosis (153.9; 46.9-505.0), hypoglycemia (30.8; 18.1-52.3), hypertension (29.1; 11.2-75.3), vitamin D deficiency (19.9; 11.1-35.5), nutritional deficiency (19.2; 12.5-29.6), iron deficiency (17.9; 9.8-32.7), iron deficiency anemia (15.5; 8.0-30.0), and anemia (11.3; 7.2-17.7) (**Supplemental Figure S1**).

In adults with GSDIa vs comparators, the most frequently diagnosed complications were hepatocellular adenoma (279.5; 95% CI, 37.8-2065.6), gastrostomy (124.0; 16.1-955.9), liver transplantation (113.3; 14.6-880.1), acidosis (32.0; 19.7-1.9), hepatomegaly (24.9; 15.6-39.5), hyperuricemia (16.6; 8.0-34.5), hypoglycemia (14.7; 9.1-23.6), anorexia (12.6; 6.3-25.1), acute kidney failure (12.3; 8.3-18.1), and pancreatitis (10.3; 4.4-23.9) (**Supplemental Figure S2**).

**Figure 3.**
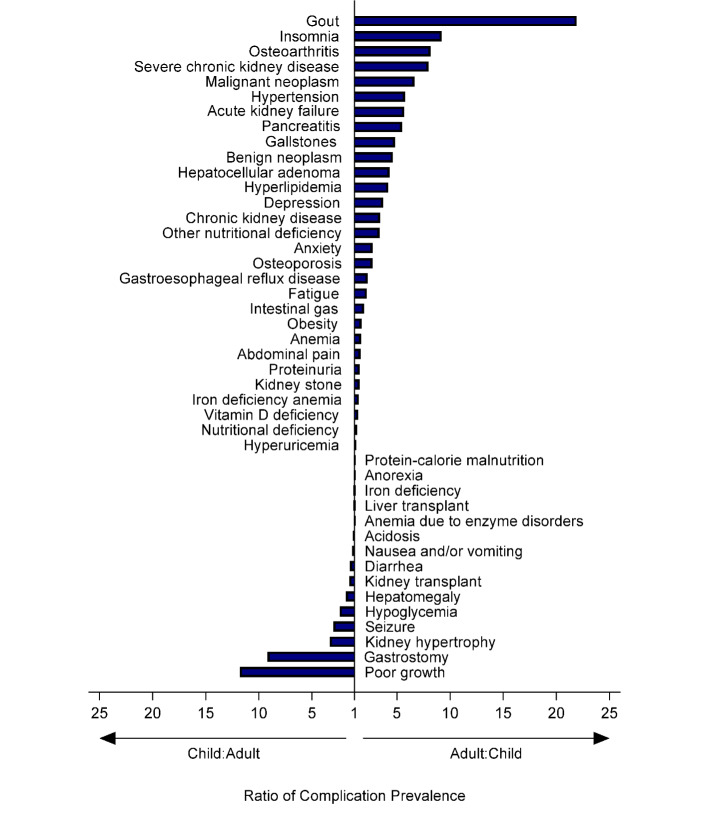
Relative Prevalence of Complications in Adults and Children With GSDIa Complication prevalence ratios were calculated as adult percentage/child percentage ratio (for complications more frequent in adults vs children) and as child percentage/adult percentage ratio (for complications more frequent in children vs adults). Due to a denominator of zero, adult/child ratios were not calculated for atherosclerotic heart disease (8.6% vs 0%), pulmonary hypertension (2.96% vs 0%), dialysis (0.81% vs 0%), and focal segmental glomerulosclerosis (0.27% vs 0%).

In general, the frequency of each type of complication increased with age in patients with GSDIa and in comparators, with the highest prevalence occurring in those aged 65 years or older (**Supplemental Figure S3, Supplemental Table S2**). Complications that occurred in adults with GSDIa, but not in children with GSDIa, were atherosclerotic heart disease (8.6% vs 0%), pulmonary hypertension (3.0% vs 0%), primary liver cancer (1.88% vs 0%), dialysis (0.8% vs 0%), and focal segmental glomerulosclerosis (0.3% vs 0%) (**Supplemental Table S2**). Complications occurring more often in adults than in children tended to reflect the chronic and progressive nature of GSDIa. The most frequent complications in adults vs children (reported as the adult/child ratio of prevalence percentages) included gout (11.8%/0.5%; ratio, 21.9), insomnia (10.0%/1.1%; ratio, 9.2), osteoarthritis (22.0%/2.7%; ratio, 8.2); severe chronic kidney disease (4.3%/0.5%; ratio, 8.0); malignant neoplasm (10.8%/1.6%; ratio, 6.6), hypertension (49.7%/8.7%; ratio, 5.8), acute kidney failure (15.3%/2.7%; ratio, 5.7), pancreatitis (3.0%/0.5%; ratio, 5.5), gallstones (7.8%/1.6%; ratio, 4.8), benign neoplasm (37.4%/8.1%; ratio, 4.6), hepatocellular adenoma (7.0%/1.6%; ratio, 4.3), and hyperlipidemia (45.2%/10.8%; ratio, 4.2) (**Figure 3**). In contrast, complications occurring more often in children than in adults tended to be acute manifestations of GSDIa. These complications included poor growth (22.2%/1.9%; ratio, 11.8), gastrostomy (29.7%/3.2%; ratio, 9.2), kidney hypertrophy (2.7%/0.8%; ratio, 3.3), seizure (1.6%/0.5%; ratio, 3.0), hypoglycemia (27.0%/11.3%; ratio, 2.4), hepatomegaly (28.7%/15.9% ratio, 1.8), kidney transplantation (1.6%/1.1%; ratio, 1.5), diarrhea (26.5%/18.6%, ratio, 1.4), nausea and/or vomiting (43.8%/35.8%; ratio, 1.2), acidosis (20.0%/17.2%; ratio, 1.2), and anemia due to enzyme disorders (43.8%/40.6%; ratio, 1.1).

### Resource Utilization Among Patients With GSDIa

Patients with GSDIa, vs comparators, had a higher mean (SD) number of annual hospital admissions (0.5 [1.1] vs 0.1 [0.3] per patient per year [PPPY]) and a longer mean (SD) average annualized length of stay (3.1 [8.4] vs 0.4 [5.2] days) (**Table 2**). They also had more mean (SD) annual emergency department visits (1.3 [2.1] vs 0.3 [0.6]), outpatient hospital visits (5.9 [8.2] vs 1.2 [3.6]), physician office visits (10.5 [12.8] vs 6.0 [7.8]), other outpatient visits (9.5 [28.0] vs 1.5 [6.2]), and pharmacy visits (12.1 [26.8] vs 5.0 [9.4] per month (**Table 2**). During the approximate 5.75-year follow-up period, a higher proportion of patients with GSDIa vs comparators had at least 1 hospitalization (255 [45.8%] vs 570 [10.2%]), only 1 hospitalization (107 [19.2%] vs 442 [7.9%]), and at least 2 hospitalizations (148 [26.6%] vs 128 [2.3%]).

**Table 2.** Healthcare Resource Utilization

	**Pediatric**		**Adult**		**Total**	
	**GSDIa (n=185)**	**Comparator (n=1850)**	**GSDIa (n=372)**	**Comparator (n=3720)**	**GSDIa (n=557)**	**Comparator (n=5570)**
Inpatient						
Patients with ≥1 hospitalization, n (%)	94 (50.8)	186 (10.1)	161 (43.3)	384 (10.3)	255 (45.8)	570 (10.2)
Mean (SD) hospitalizations PPPY, n	0.6 (1.2)	0.1 (0.2)	0.5 (1.1)	0.1 (0.3)	0.5 (1.1)	0.1 (0.3)
Mean (SD) length of stay per hospitalization PPPY, days	2.7 (6.6)	0.2 (1.5)	3.2 (9.2)	0.4 (6.2)	3.1 (8.4)	0.4 (5.2)
Mean (SD) cost per hospitalization ($)	29 510 (41 930)	16 120 (42 230)	33 910 (78 280)	18 250 (27 240)	32 200 (66 550)	17 690 (31 880)
Emergency department visits						
Patients with a visit, n (%)	129 (69.7)	557 (30.1)	258 (69.4)	1256 (33.8)	387 (69.5)	1813 (32.6)
Visits PPPY, n	1.2 (1.8)	0.2 (0.5)	1.3 (2.3)	0.3 (0.7)	1.3 (2.1)	0.3 (0.6)
Mean (SD) cost per visit ($)	7400 (21 430)	1430 (9370)	7230 (39 560)	2610 (11 370)	7290 (34 680)	2290 (10 870)
Outpatient hospital visits						
Patients with a visit, n (%)	163 (88.1)	849 (45.9)	338 (90.9)	2230 (60.0)	501 (90.0)	3079 (55.3)
Visits PPPY, n	5.8 (9.2)	0.6 (1.5)	5.9 (7.7)	1.6 (4.2)	5.9 (8.2)	1.2 (3.6)
Mean (SD) cost per visit ($)	1030 (2150)	630 (2220)	1030 (4890)	790 (2710)	1030 (4170)	770 (2630)
Physician office visits						
Patients with a visit, n (%)	179 (96.8)	1786 (96.5)	359 (96.5)	3493 (93.9)	538 (96.6)	5279 (94.8)
Visits PPPY, n	10.8 (16.2)	5.4 (6.4)	10.4 (10.7)	6.3 (8.3)	10.5 (12.8)	6.0 (7.8)
Mean (SD) cost per visit ($)	170 (230)	140 (180)	150 (340)	110 (200)	160 (310)	120 (200)
Other outpatient visits						
Patients with a visit, n (%)	159 (86.0)	929 (50.2)	326 (87.6)	2417 (65.0)	485 (87.1)	3346 (60.1)
Visits PPPY, n	15.0 (42.2)	1.0 (4.6)	6.7 (16.3)	1.8 (6.9)	9.5 (28.0)	1.5 (6.2)
Mean (SD) cost per visit ($)	410 (1550)	200 (650)	300 (2870)	270 (1360)	350 (2280)	250 (1250)
Pharmacy visitsa						
Patients with a pharmacy visit, n (%)	146 (78.9)	1198 (64.8)	331 (89.0)	2850 (76.6)	477 (85.6)	4048 (72.7)
Pharmacy visits PPPY, n	11.7 (39.4)	2.1 (4.0)	12.2 (17.6)	6.4 (10.9)	12.1 (26.8)	5.0 (9.4)
Mean (SD) cost per visit ($)	460 (1240)	100 (370)	450 (2020)	180 (1110)	450 (1780)	170 (1040)

Abbreviations: GSDIa, glycogen storage disease type Ia; PPPY, per patient per year.

^a^One pharmaceutical visit (ie, one pharmacy record) of the same date may have had multiple prescription fills.

Across age groups, infants most often had only 1 hospitalization (GSDIa, 39.3%; comparator, 49.6%), whereas 2 or more hospitalizations most often occurred in older adults (GSDIa, 47.83%; comparator, 12.17%), followed by infants (GSDIa, 42.86%; comparator, 3.57%) (**Supplemental Figure S4**). Adults with GSDIa, compared with children with GSDIa, had a slightly longer mean length of hospitalization stay (3.23 vs 2.74 days), slightly more pharmacy visits (12.2 vs 11.7 PPPY), and slightly fewer physician office visits (10.4 vs 10.8 PPPY). They had similar frequencies of hospitalizations (0.5 vs 0.6 PPPY), emergency department visits (1.3 vs 1.2 PPPY), and outpatient hospital visits (5.9 vs 5.8 PPPY) (**Table 2**).

### Healthcare Costs Among Patients With GSDIa

**Figure 4.**
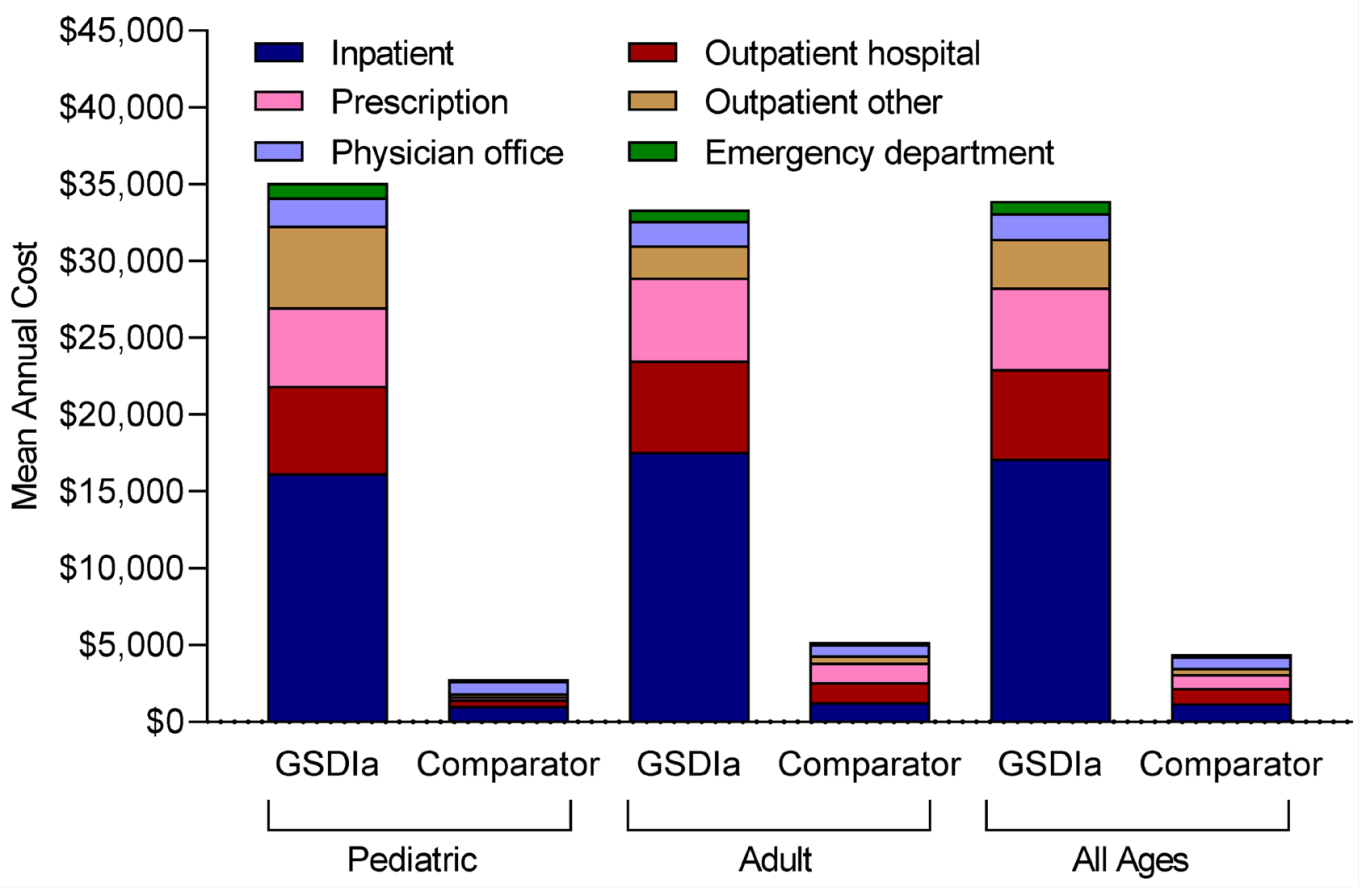
Mean Annual Healthcare Costs per Patient by Setting of Care Abbreviation: GSDIa, glycogen storage disease type Ia.

Patients with GSDIa, vs comparators, had higher mean (SD) annual total healthcare costs ($33 910 [$75 660] vs $4410 [$19 120]), including inpatient costs ($17 100 [$55 220] vs $1210 [$10 770]), outpatient hospital costs ($5860 [$13 760] vs $1010 [$6370]), pharmacy costs ($5320 [$18 210] vs $910 [$7070]), other outpatient costs ($3160 [$11 380] vs $400 [$3350]), physician office costs ($1680 [$2780] vs $750 [$1250]), and emergency department costs ($800 [$1740] vs $150 [$500]) (**Figure 4**). Similarly, patients with GSDIa vs comparators had higher costs per hospital admission ($32 200 [$66 550] vs $17 690 [$31 880]); costs per emergency department visit ($7290 [$34 680] vs $2290 [$10 870]); costs per outpatient hospital visit ($1030 [$4170] vs $770 [$2630]), costs per pharmacy visit ($450 [$1780] vs $170 [$1040]), costs per other outpatient visit ($350 [$2280] vs $250 [$1250]), and costs per physician office visit ($160 [$310] vs $120 [$200]) (**Table 2**).

Adults with GSDIa, compared with children with GSDIa, had lower other outpatient costs ($2100 [$8880] vs $5310 [$15 000]), slightly lower mean (SD) annual total healthcare costs ($27 920 [$73 260] vs $2  960 [$59 804]), physician office costs ($1590 [$2780] vs $1850 [$2790]), and emergency department costs ($730 [$1420] vs $940 [$2250]), but had slightly higher inpatient costs ($17 570 [$60 340] vs $16 150 [$43 250]) and pharmacy costs ($5410 [$17 800] vs $5130 [$19 060]) (**Figure 4**). Adult patients with GSDIa vs children with GSDIa had greater mean (SD) costs per hospital admission ($33 910 [$78 280] vs $29 510 [$41 930]), slightly lower costs per emergency department visit ($7230 [$39 560] vs $7400 [$21 430]), physician office visit ($150 [$340] vs $170 [$230]), other outpatient visit ($300 [$2870] vs 410 [$1550]), and pharmacy visit ($450 [$2020] vs $460 [$1240]), and similar costs per outpatient hospital visit ($1030 [$4890] vs $1030 [$2150]) (**Table 2**).

## DISCUSSION

Although patient monitoring and dietary management in recent decades have improved the prognosis for patients with GSDIa overall, maintaining glycemic control is challenging, and long-term complications may persist.^8,11,12,17,19–22,27^ This study adds to existing knowledge by providing real-world evidence on the comprehensive burden of GSDIa, both in terms of clinical complications and healthcare resource utilization, which has been insufficiently quantified in previous research. This large, retrospective, real-world database analysis demonstrated that GSDIa was associated with a substantial burden of illness for patients, with a high prevalence of acute, chronic, and often serious complications related not only to the underlying pathophysiology of the disease, but also likely related to dietary management strategies (**Supplemental Table S3**). By quantifying the healthcare resource utilization and costs associated with these complications, this study expands on earlier reports that have focused primarily on clinical outcomes. Consistent with prior reports, frequent complications included liver and/or kidney transplant, hematologic complications (eg, acidosis and anemia), renal complications (eg, hyperuricemia, acute kidney failure, and chronic kidney disease), nutritional complications (eg, gastrostomy, anorexia, and protein-calorie malnutrition), digestive system complications (eg, pancreatitis), hepatic complications (eg, hepatomegaly and hypoglycemia), and neoplasms like hepatocellular adenoma.^3,5–7,16,17,28–30^

Because UCCS supplementation does not address the underlying disease and may trigger secondary metabolic manifestations in patients with GSDIa, chronic and serious complications persist, requiring lifelong care and resulting in increased mortality.^4,12–18^ In this study, the complications observed among patients with GSDIa demonstrate the chronic and progressive nature of GSDIa. This study’s findings also underscore the significant healthcare burden associated with managing these complications, highlighting the need for improved treatment options. The adults with GSDIa more often than children experienced complications common in advanced stages of disease, including pulmonary hypertension, hyperlipidemia, malignant and benign neoplasms (eg, primary liver cancer and hepatocellular adenoma), dialysis; focal segmental glomerulosclerosis, gout, osteoarthritis, osteoporosis, chronic kidney disease, kidney failure, pancreatitis, and gallstones.^3,5–7,16,17,28–32^ In contrast, children with GSDIa exhibited complications of GSDIa that are typically acute manifestations resulting from suboptimal metabolic control, such as poor growth, hepatomegaly, lactic acidosis, hypoglycemia, hypertension, vitamin D deficiency, nutritional deficiency, iron deficiency, anemia, and seizures.^6,7,17^ The differentiation in complication profiles between adults and children emphasizes the need for treatment that addresses the underlying cause of the disease to prevent both acute complications and disease progression. Children with GSDIa were also more likely than adults with GSDIa to experience renal complications (especially kidney hypertrophy) and certain digestive complications (eg, diarrhea and nausea and/or vomiting).

Gastrostomy occurred frequently among children with GSDIa, reflecting current guidelines that include nasogastric tubes or gastrostomy tubes for overnight dietary supplementation with formula in infants.^8^ Patient monitoring combined with supplementation with carbohydrates such as UCCS are the mainstay of disease management among children and adults with GSDIa.^8,17^ Although data on dietary supplementation were not available, the prevalence of digestive complications (eg, intestinal gas, nausea/vomiting, GERD, and diarrhea), hyperlipidemia, lactic acidosis, and obesity experienced among children and adults suggest that supplementation with UCCS was common in the study population. Dietary supplementation with low-digestible carbohydrates such as UCCS is associated with abdominal pain, intestinal gas, and diarrhea,^19–21,23,33^ and worsened lactic acidosis and weight gain are possible.^8^

In our study, patients with GSDIa had substantial healthcare resource utilization vs comparators, requiring more hospitalizations (45.8% vs 10.2%; 0.53 vs 0.06 hospitalizations PPPY), including 2 or more hospitalizations (26.6% vs 2.3%), and longer length of stay (3.1 vs 0.4 days). Multiple hospitalizations were common among adults, again underscoring the serious and progressive nature of complications in GSDIa, and among infants, when metabolic control is most challenging due predominantly to the difficulty in feeding.^8,22^ This study is the first to quantify healthcare utilization and costs in this population, demonstrating that mean annual total healthcare costs for patients with GSDIa were almost 8 times higher than those of comparators ($33 910 vs $4410). These costs were driven primarily by hospitalizations, outpatient hospital visits, and prescriptions.

### Limitations

This analysis had several limitations. The results may be subject to measurement errors, and medical claims may have been driven by reimbursement concerns that may not accurately reflect the medical condition. Additionally, the retrospective design limited information to encounters where an insurance claim was generated and may not have captured less serious complications. Given that patient counts of 1 to 10 were not reported for privacy reasons, the analysis captured complication ICD-10 codes occurring in any billing position to be less restrictive, especially for rare but serious complications. Furthermore, it is possible that the prevalence of some conditions may be modestly overestimated as a result of bill practices. However, because the analysis used a matched comparator group, the ORs should have been unaffected.

Dietary management is not captured in claims data. Therefore, this analysis could not evaluate the safety or efficacy of dietary management strategies among patients with GSDIa. As dietary management is the current standard of care for patients with GSDIa, it is likely that this was prevalent in the study cohort. Finally, quality of life and mortality were not captured in this study.

### Implications

Our study has important implications for both clinical practice and healthcare policy. We found that GSDIa is associated with a high burden of illness and significant healthcare resource utilization, including frequent hospitalizations, long lengths of stay, and elevated healthcare costs. Clinically, these real-world findings underscore the inadequacy of the current standard of care and the need to support the development of therapies for GSDIa. Consistent with previous data showing that acute metabolic complications occur despite dietary management,^34^ there are no approved treatments that address the underlying disease pathophysiology.^17^ Personalized and age-specific treatment approaches are particularly important given the differing complication profiles between adults and children with GSDIa. Early interventions targeting acute complications in children, and specialized care for chronic, progressive conditions in adults, could help mitigate the disease burden.

From a policy perspective, the substantial cost differences between patients with GSDIa and comparators highlight the need for better resource allocation, increased access to multidisciplinary care, and potential reimbursement policies that account for the lifelong needs of this population. When compared with other rare genetic disorders,^35^ the healthcare costs and resource utilization associated with GSDIa are similarly high, underscoring the broader need for rare disease–specific policies that incentivize the development of new therapies.^36^ These findings can inform policymakers as they consider coverage and pricing strategies for innovative treatments that can lessen patient burden and reduce the economic strain on healthcare systems.

## CONCLUSIONS

This large, real-world analysis of GSDIa provides comprehensive insights into the clinical complications, healthcare resource utilization, and economic burden associated with managing this rare disease. The study confirms the substantial illness burden for GSDIa patients and highlights the high prevalence of serious complications, especially in adult patients. Moreover, it quantifies the significant healthcare costs incurred, driven largely by hospitalizations and outpatient care, revealing that patients with GSDIa incur almost eight times the costs of matched comparators.

The implications of these findings are far-reaching. There is a significant unmet need for treatments that address the underlying metabolic dysfunction, as current UCCS-based dietary management only mitigates symptoms and is associated with significant long-term complications such as hyperlipidemia, lactic acidosis, and obesity. Future research should focus on developing novel therapies that can target the root cause of the disease.

For healthcare policymakers, the results support the urgency of prioritizing rare diseases like GSDIa in resource allocation and reimbursement policies, especially in light of the financial impact on healthcare systems. Future studies should also investigate the broader implications of GSDIa management, including comparisons to other rare genetic disorders, to inform strategies that improve patient outcomes while managing healthcare resource utilization. These findings highlight the need for more effective treatments and management approaches that go beyond current dietary interventions, ensuring that patients with GSDIa receive optimal care throughout their lifetime.

### Availability of Data and Materials

The data sets used and analyzed in this study are available from the corresponding author on reasonable request.

### Competing Interests

Eliza Kruger, Justin Nedzesky, and Nina Thomas were employees of Ultragenyx Pharmaceutical Inc. at the time the work was conducted. Jeffrey D. Dunn is a consultant for Ultragenyx Pharmaceutical Inc. Andrew Grimm is an employee of and shareholder in Ultragenyx Pharmaceutical Inc.

## Supplementary Material

Online Supplementary Material
