# Supplementary material for: Glycogen Storage Disease Type Ia: A Retrospective Claims Analysis of Complications, Resource Utilization, and Cost of Care
Source: J Health Econ Outcomes Res. 2025 Jan 7;12(1):13–21. doi: 10.36469/001c.125886 (PMC11716495; doi:10.36469/001c.125886)
Supplement: Online Supplementary Material [file jheor_2025_12_1_125886_261028.pdf]

Journal of Health Economics and Outcomes Research

Online Supplementary Material

Glycogen Storage Disease Type Ia: A Retrospective Claims Analysis of Complications, Resource Utilization, and Cost of Care. *JHEOR*. 2025;12(1):13-21. doi:10.36469/jheor.2025.125886

Table S1: ICD-10-CM Codes Used to Identify Complications in Patients With GSDIa

Table S2: Prevalence of Complications

Table S3: Possible Complication Etiologies

Figure S1: Complications in Children With GSDIa vs Comparators

Figure S2: Complications in Adults With GSDIa vs Comparators

Figure S3: Frequency of Complication Types by Patient Age in Patients With GSDIa (A) and in Comparators (B)

Figure S4: Frequency of Hospitalizations by Patient Age in Patients With GSDIa (A) and in Comparators (B)

This supplementary material has been provided by the authors to give readers additional information about their work.

This is an open-access article distributed under the terms of the Creative Commons Attribution 4.0 International License (CCBY-4.0). View this license's legal deed at http://creativecommons.org/licenses/by/4.0 and legal code at http://creativecommons.org/licenses/by/4.0/legalcode for more information.

Table S1. ICD-10-CM Codes Used to Identify Complications in Patients With GSDIa

Complication	ICD-10-D Codes
**GSDIa**	**ICD-10-D-E7401, excluding patients with inflammatory bowel disease**
Digestive System	
Abdominal Pain	ICD-10-D-R1010, ICD-10-D-R1011, ICD-10-D-R1012, ICD-10-D-R1013, ICD-10-D-R1030, ICD-10-D-R1031, ICD-10-D-R1032, ICD-10-D-R1033, ICD-10-D-R1084, ICD-10-D-R109
Diarrhea	ICD-10-D-R197
Gallstones	ICD-10-D-K8000, ICD-10-D-K8001, ICD-10-D-K8010, ICD-10-D-K8011, ICD-10-D-K8012, ICD-10-D-K8013, ICD-10-D-K8018, ICD-10-D-K8019, ICD-10-D-K8020, ICD-10-D-K8021, ICD-10-D-K8030, ICD-10-D-K8031, ICD-10-D-K8032, ICD-10-D-K8033, ICD-10-D-K8034, ICD-10-D-K8035, ICD-10-D-K8037, ICD-10-D-K8040, ICD-10-D-K8041, ICD-10-D-K8042, ICD-10-D-K8043, ICD-10-D-K8044, ICD-10-D-K8045, ICD-10-D-K8046, ICD-10-D-K8047, ICD-10-D-K8050, ICD-10-D-K8051, ICD-10-D-K8060, ICD-10-D-K8061, ICD-10-D-K8062, ICD-10-D-K8063, ICD-10-D-K8064, ICD-10-D-K8065, ICD-10-D-K8066, ICD-10-D-K8067, ICD-10-D-K8070, ICD-10-D-K8071, ICD-10-D-K8080, ICD-10-D-K8081
Gastroesophageal reflux disease	ICD-10-D-K210, ICD-10-D-K219
Intestinal gas	ICD-10-D-R140, ICD-10-D-R141, ICD-10-D-R142, ICD-10-D-R143
Nausea and/or Vomiting	ICD-10-D-R110, ICD-10-D-R1110, ICD-10-D-R1111, ICD-10-D-R1112, ICD-10-D-R112
Pancreatitis	ICD-10-D-K850, ICD-10-D-K8500, ICD-10-D-K8501, ICD-10-D-K8502, ICD-10-D-K851, ICD-10-D-K8510, ICD-10-D-K8511, ICD-10-D-K8512, ICD-10-D-K852, ICD-10-D-K8520, ICD-10-D-K8521, ICD-10-D-K8522, ICD-10-D-K853, ICD-10-D-K8530, ICD-10-D-K8531, ICD-10-D-K8532, ICD-10-D-K858, ICD-10-D-K8580, ICD-10-D-K8581, ICD-10-D-K8582, ICD-10-D-K859, ICD-10-D-K8590, ICD-10-D-K8591, ICD-10-D-K8592, ICD-10-D-K860, ICD-10-D-K861
Nutritional	
Anorexia	ICD-10-D-R630
Gastrostomy	ICD-10-D-K9420, ICD-10-D-K9421, ICD-10-D-K9422, ICD-10-D-K9423, ICD-10-D-K9429, ICD-10-D-Z431, ICD-10-D-Z931
Iron deficiency	ICD-10-D-D500, ICD-10-D-D508, ICD-10-D-D509, ICD-10-D-E611
Nutritional deficiency	ICD-10-D-E559, ICD-10-D-D500, ICD-10-D-D508, ICD-10-D-D509, ICD-10-D-E611, ICD-10-D-D530, ICD-10-D-E40, ICD-10-D-E41, ICD-10-D-E42, ICD-10-D-E43, ICD-10-D-E440, ICD-10-D-E441, ICD-10-D-E45, ICD-10-D-E46, ICD-10-D-E640, ICD-10-D-E500, ICD-10-D-E501, ICD-10-D-E502, ICD-10-D-E505, ICD-10-D-E506, ICD-10-D-E507, ICD-10-D-E508, ICD-10-D-E509, ICD-10-D-E5111, ICD-10-D-E5112, ICD-10-D-E512, ICD-10-D-E518, ICD-10-D-E519, ICD-10-D-E52, ICD-10-D-E530, ICD-10-D-E531, ICD-10-D-E538, ICD-10-D-E539, ICD-10-D-E54, ICD-10-D-E560, ICD-10-D-E561, ICD-10-D-E568, ICD-10-D-E569, ICD-10-D-E58, ICD-10-D-E59, ICD-10-D-E60, ICD-10-D-E630, ICD-10-D-E631, ICD-10-D-E638, ICD-10-D-E639, ICD-10-D-E641, ICD-10-D-E642, ICD-10-D-E648, ICD-10-D-E649, ICD-10-D-D511, ICD-10-D-D513, ICD-10-D-D518, ICD-10-D-D519, ICD-10-D-D520, ICD-10-D-D528, ICD-10-D-D529
Obesity	ICD-10-D-E6601, ICD-10-D-E6609, ICD-10-D-E662, ICD-10-D-E668, ICD-10-D-E669, ICD-10-D-Z6830, ICD-10-D-Z6831, ICD-10-D-Z6832,

Complication	ICD-10-D Codes
	ICD-10-D-Z6833, ICD-10-D-Z6834, ICD-10-D-Z6835, ICD-10-D-Z6836, ICD-10-D-Z6837, ICD-10-D-Z6838, ICD-10-D-Z6839, ICD-10-D-Z6841, ICD-10-D-Z6842, ICD-10-D-Z6843, ICD-10-D-Z6844, ICD-10-D-Z6845, ICD-10-D-Z6854
Other nutritional deficiency	ICD-10-D-E500, ICD-10-D-E501, ICD-10-D-E502, ICD-10-D-E505, ICD-10-D-E506, ICD-10-D-E507, ICD-10-D-E508, ICD-10-D-E509, ICD-10-D-E5111, ICD-10-D-E5112, ICD-10-D-E512, ICD-10-D-E518, ICD-10-D-E519, ICD-10-D-E52, ICD-10-D-E530, ICD-10-D-E531, ICD-10-D-E538, ICD-10-D-E539, ICD-10-D-E54, ICD-10-D-E560, ICD-10-D-E561, ICD-10-D-E568, ICD-10-D-E569, ICD-10-D-E58, ICD-10-D-E59, ICD-10-D-E60, ICD-10-D-E630, ICD-10-D-E631, ICD-10-D-E638, ICD-10-D-E639, ICD-10-D-E641, ICD-10-D-E642, ICD-10-D-E648, ICD-10-D-E649, ICD-10-D-D511, ICD-10-D-D513, ICD-10-D-D518, ICD-10-D-D519, ICD-10-D-D520, ICD-10-D-D528, ICD-10-D-D529
Poor growth	ICD-10-D-R6251, ICD-10-D-R627, ICD-10-D-R620, ICD-10-D-R6250, ICD-10-D-R6252, ICD-10-D-R6259
Protein-calorie malnutrition	ICD-10-D-D530, ICD-10-D-E40, ICD-10-D-E41, ICD-10-D-E42, ICD-10-D-E43, ICD-10-D-E440, ICD-10-D-E441, ICD-10-D-E45, ICD-10-D-E46, ICD-10-D-E640
Vitamin D deficiency	ICD-10-D-E559
Hematologic	
Anemia	ICD-10-D-D500, ICD-10-D-D508, ICD-10-D-D509, ICD-10-D-D510, ICD-10-D-D511, ICD-10-D-D513, ICD-10-D-D518, ICD-10-D-D519, ICD-10-D-D520, ICD-10-D-D528, ICD-10-D-D529, ICD-10-D-D530, ICD-10-D-D531, ICD-10-D-D532, ICD-10-D-D538, ICD-10-D-D539, ICD-10-D-D630, ICD-10-D-D631, ICD-10-D-D638, ICD-10-D-D641, ICD-10-D-D643, ICD-10-D-D6481, ICD-10-D-D6489, ICD-10-D-D649
Anemia due to enzyme disorders	ICD-10-D-D550, ICD-10-D-D551, ICD-10-D-D552, ICD-10-D-D553, ICD-10-D-D558, ICD-10-D-D559
Iron deficiency anemia	ICD-10-D-D500, ICD-10-D-D508, ICD-10-D-D509
Acidosis	ICD-10-D-E872
Cardiovascular/cardiopulmonary	
Atherosclerotic heart disease	ICD-10-D-I2510, ICD-10-D-I25110, ICD-10-D-I25111, ICD-10-D-I25118, ICD-10-D-I25119
Hypertension	ICD-10-D-I10, ICD-10-D-I110, ICD-10-D-I119, ICD-10-D-I120, ICD-10-D-I129, ICD-10-D-I130, ICD-10-D-I131, ICD-10-D-I1310, ICD-10-D-I1311, ICD-10-D-I132, ICD-10-D-I150, ICD-10-D-I151, ICD-10-D-I152, ICD-10-D-I158, ICD-10-D-I159
Pulmonary hypertension	ICD-10-D-I270, ICD-10-D-I272, ICD-10-D-I2720, ICD-10-D-I2721, ICD-10-D-I2722, ICD-10-D-I2729
Hepatic	
Hypoglycemia	ICD-10-D-E08641, ICD-10-D-E08649, ICD-10-D-E10641, ICD-10-D-E10649, ICD-10-D-E11641, ICD-10-D-E11649, ICD-10-D-E13641, ICD-10-D-E13649, ICD-10-D-E15, ICD-10-D-E160, ICD-10-D-E161, ICD-10-D-E162, ICD-10-D-P704
Hepatomegaly	ICD-10-D-R160, ICD-10-D-R162
Hyperlipidemia	ICD-10-D-E780, ICD-10-D-E7800, ICD-10-D-E781, ICD-10-D-E782, ICD-10-D-E784, ICD-10-D-E785
Musculoskeletal	

Complication	ICD-10-D Codes
Fatigue	ICD-10-D-R530, ICD-10-D-R531, ICD-10-D-R538, ICD-10-D-R5381, ICD-10-D-R5382, ICD-10-D-R5383
Gout	ICD-10-D-M1000, ICD-10-D-M10011, ICD-10-D-M10012, ICD-10-D-M10021, ICD-10-D-M10031, ICD-10-D-M10032, ICD-10-D-M10039, ICD-10-D-M10041, ICD-10-D-M10042, ICD-10-D-M10061, ICD-10-D-M10062, ICD-10-D-M10069, ICD-10-D-M10071, ICD-10-D-M10072, ICD-10-D-M10079, ICD-10-D-M1009, ICD-10-D-M1030, ICD-10-D-M10332, ICD-10-D-M10361, ICD-10-D-M10362, ICD-10-D-M10371, ICD-10-D-M10372, ICD-10-D-M10379, ICD-10-D-M1039, ICD-10-D-M1040, ICD-10-D-M10461, ICD-10-D-M10462, ICD-10-D-M10471, ICD-10-D-M10472, ICD-10-D-M10479, ICD-10-D-M1049, ICD-10-D-M109, ICD-10-D-M1A00X0, ICD-10-D-M1A00X1, ICD-10-D-M1A0410, ICD-10-D-M1A0411, ICD-10-D-M1A0420, ICD-10-D-M1A0610, ICD-10-D-M1A0611, ICD-10-D-M1A0620, ICD-10-D-M1A0621, ICD-10-D-M1A0710, ICD-10-D-M1A0711, ICD-10-D-M1A0720, ICD-10-D-M1A0721, ICD-10-D-M1A0790, ICD-10-D-M1A09X0, ICD-10-D-M1A09X1, ICD-10-D-M1A30X0, ICD-10-D-M1A30X1, ICD-10-D-M1A3120, ICD-10-D-M1A3191, ICD-10-D-M1A3690, ICD-10-D-M1A3710, ICD-10-D-M1A3721, ICD-10-D-M1A38X1, ICD-10-D-M1A39X0, ICD-10-D-M1A39X1, ICD-10-D-M1A40X0, ICD-10-D-M1A4291, ICD-10-D-M1A4710, ICD-10-D-M1A4711, ICD-10-D-M1A4790, ICD-10-D-M1A9XX0, ICD-10-D-M1A9XX1
Osteoarthritis	ICD-10-D-M160, ICD-10-D-M1610, ICD-10-D-M1611, ICD-10-D-M1612, ICD-10-D-M162, ICD-10-D-M1630, ICD-10-D-M1631, ICD-10-D-M1632, ICD-10-D-M166, ICD-10-D-M167, ICD-10-D-M169, ICD-10-D-M170, ICD-10-D-M1710, ICD-10-D-M1711, ICD-10-D-M1712, ICD-10-D-M174, ICD-10-D-M175, ICD-10-D-M179, ICD-10-D-M180, ICD-10-D-M1810, ICD-10-D-M1811, ICD-10-D-M1812, ICD-10-D-M184, ICD-10-D-M1851, ICD-10-D-M1852, ICD-10-D-M189, ICD-10-D-M19011, ICD-10-D-M19012, ICD-10-D-M19019, ICD-10-D-M19021, ICD-10-D-M19022, ICD-10-D-M19029, ICD-10-D-M19031, ICD-10-D-M19032, ICD-10-D-M19039, ICD-10-D-M19041, ICD-10-D-M19042, ICD-10-D-M19049, ICD-10-D-M19071, ICD-10-D-M19072, ICD-10-D-M19079, ICD-10-D-M19211, ICD-10-D-M19212, ICD-10-D-M19219, ICD-10-D-M19221, ICD-10-D-M19222, ICD-10-D-M19231, ICD-10-D-M19241, ICD-10-D-M19242, ICD-10-D-M19249, ICD-10-D-M19271, ICD-10-D-M19272, ICD-10-D-M1990, ICD-10-D-M1991, ICD-10-D-M1993
Osteoporosis	ICD-10-D-M8000XA, ICD-10-D-M8000XD, ICD-10-D-M8000XG, ICD-10-D-M8000XP, ICD-10-D-M8000XS, ICD-10-D-M80019D, ICD-10-D-M80021K, ICD-10-D-M80021S, ICD-10-D-M80022A, ICD-10-D-M80022G, ICD-10-D-M80022S, ICD-10-D-M80031A, ICD-10-D-M80051A, ICD-10-D-M80051D, ICD-10-D-M80051K, ICD-10-D-M80051S, ICD-10-D-M80052A, ICD-10-D-M80052D, ICD-10-D-M80059A, ICD-10-D-M80059S, ICD-10-D-M80061D, ICD-10-D-M80062A, ICD-10-D-M80071D, ICD-10-D-M80072A, ICD-10-D-M8008XA, ICD-10-D-M8008XD, ICD-10-D-M8008XG, ICD-10-D-M8008XS, ICD-10-D-M8080XA, ICD-10-D-M8080XD, ICD-10-D-M8080XG, ICD-10-D-M8080XK, ICD-10-D-M8080XS, ICD-10-D-M80811A, ICD-10-D-M80811D, ICD-10-D-M80812A, ICD-10-D-M80821A, ICD-10-D-M80821K, ICD-10-D-M80822A, ICD-10-D-M80822D, ICD-10-D-M80822K, ICD-10-D-M80832A, ICD-10-D-M80851A, ICD-10-D-M80851D, ICD-10-D-M80851S, ICD-10-D-

Complication	ICD-10-D Codes
	M80852A, ICD-10-D-M80852D, ICD-10-D-M80852S, ICD-10-D-M80859A, ICD-10-D-M80861A, ICD-10-D-M80862D, ICD-10-D-M80872A, ICD-10-D-M8088XA, ICD-10-D-M8088XD, ICD-10-D-M8088XG, ICD-10-D-M8088XS, ICD-10-D-M810, ICD-10-D-M816, ICD-10-D-M818
Psychiatric/neurologic	
Anxiety	ICD-10-D-F064, ICD-10-D-F411, ICD-10-D-F413, ICD-10-D-F418, ICD-10-D-F419, ICD-10-D-F4322, ICD-10-D-F4323
Depression	ICD-10-D-F320, ICD-10-D-F321, ICD-10-D-F322, ICD-10-D-F323, ICD-10-D-F324, ICD-10-D-F325, ICD-10-D-F328, ICD-10-D-F3289, ICD-10-D-F329, ICD-10-D-F330, ICD-10-D-F331, ICD-10-D-F332, ICD-10-D-F333, ICD-10-D-F3340, ICD-10-D-F3341, ICD-10-D-F3342, ICD-10-D-F338, ICD-10-D-F339, ICD-10-D-F4321, ICD-10-D-F4323
Insomnia	ICD-10-D-F5101, ICD-10-D-F5102, ICD-10-D-F5103, ICD-10-D-F5104, ICD-10-D-F5105, ICD-10-D-F5109, ICD-10-D-G4700, ICD-10-D-G4701, ICD-10-D-G4709, ICD-10-D-Z73810, ICD-10-D-Z73812, ICD-10-D-Z73819
Seizure	ICD-10-D-G4089
Renal	
Acute kidney failure	ICD-10-D-N170, ICD-10-D-N171, ICD-10-D-N172, ICD-10-D-N178, ICD-10-D-N179
Chronic kidney disease	ICD-10-D-N181, ICD-10-D-N182, ICD-10-D-N183, ICD-10-D-N184, ICD-10-D-N185, ICD-10-D-N186, ICD-10-D-N189, ICD-10-D-N186, ICD-10-D-Z992
Dialysis	ICD-10-D-Z4901, ICD-10-D-Z4902, ICD-10-D-Z4931, ICD-10-D-Z4932
Focal segmental glomerulosclerosis	ICD-10-D-N001, ICD-10-D-N011, ICD-10-D-N021, ICD-10-D-N031, ICD-10-D-N041, ICD-10-D-N051, ICD-10-D-N061, ICD-10-D-N071
Hyperuricemia	ICD-10-D-E790
Kidney hypertrophy	ICD-10-D-N2881
Kidney stone	ICD-10-D-N132, ICD-10-D-N200, ICD-10-D-N201, ICD-10-D-N202, ICD-10-D-N209, ICD-10-D-N210, ICD-10-D-N211, ICD-10-D-N218, ICD-10-D-N219, ICD-10-D-N22, ICD-10-D-N23
Proteinuria	ICD-10-D-R801, ICD-10-D-R809
Severe chronic kidney disease	ICD-10-D-N184, ICD-10-D-N185, ICD-10-D-N186, ICD-10-D-Z992
Neoplasm	
Benign neoplasm	ICD-10-D-D100, ICD-10-D-D101, ICD-10-D-D1030, ICD-10-D-D1039, ICD-10-D-D104, ICD-10-D-D105, ICD-10-D-D106, ICD-10-D-D107, ICD-10-D-D109, ICD-10-D-D110, ICD-10-D-D117, ICD-10-D-D119, ICD-10-D-D120, ICD-10-D-D121, ICD-10-D-D122, ICD-10-D-D123, ICD-10-D-D124, ICD-10-D-D125, ICD-10-D-D126, ICD-10-D-D127, ICD-10-D-D128, ICD-10-D-D129, ICD-10-D-D130, ICD-10-D-D131, ICD-10-D-D132, ICD-10-D-D1330, ICD-10-D-D1339, ICD-10-D-D134, ICD-10-D-D135, ICD-10-D-D136, ICD-10-D-D137, ICD-10-D-D139, ICD-10-D-D140, ICD-10-D-D141, ICD-10-D-D142, ICD-10-D-D1430, ICD-10-D-D1431, ICD-10-D-D1432, ICD-10-D-D144, ICD-10-D-D150, ICD-10-D-D151, ICD-10-D-D152, ICD-10-D-D159, ICD-10-D-D1600, ICD-10-D-D1601, ICD-10-D-D1602, ICD-10-D-D1612, ICD-10-D-

Complication	ICD-10-D Codes
	D1620, ICD-10-D-D1621, ICD-10-D-D1622, ICD-10-D-D1631, ICD-10-D-D1632, ICD-10-D-D164, ICD-10-D-D165, ICD-10-D-D166, ICD-10-D-D167, ICD-10-D-D168, ICD-10-D-D169, ICD-10-D-D170, ICD-10-D-D171, ICD-10-D-D1720, ICD-10-D-D1721, ICD-10-D-D1722, ICD-10-D-D1723, ICD-10-D-D1724, ICD-10-D-D1730, ICD-10-D-D1739, ICD-10-D-D175, ICD-10-D-D176, ICD-10-D-D1771, ICD-10-D-D1772, ICD-10-D-D1779, ICD-10-D-D179, ICD-10-D-D1800, ICD-10-D-D1801, ICD-10-D-D1802, ICD-10-D-D1803, ICD-10-D-D1809, ICD-10-D-D181, ICD-10-D-D190, ICD-10-D-D191, ICD-10-D-D197, ICD-10-D-D200, ICD-10-D-D201, ICD-10-D-D210, ICD-10-D-D2110, ICD-10-D-D2111, ICD-10-D-D2112, ICD-10-D-D2120, ICD-10-D-D2121, ICD-10-D-D2122, ICD-10-D-D214, ICD-10-D-D215, ICD-10-D-D216, ICD-10-D-D219, ICD-10-D-D220, ICD-10-D-D2211, ICD-10-D-D2212, ICD-10-D-D22122, ICD-10-D-D2221, ICD-10-D-D2222, ICD-10-D-D2230, ICD-10-D-D2239, ICD-10-D-D224, ICD-10-D-D225, ICD-10-D-D2260, ICD-10-D-D2261, ICD-10-D-D2262, ICD-10-D-D2270, ICD-10-D-D2271, ICD-10-D-D2272, ICD-10-D-D229, ICD-10-D-D230, ICD-10-D-D2310, ICD-10-D-D2311, ICD-10-D-D23111, ICD-10-D-D23112, ICD-10-D-D2312, ICD-10-D-D23121, ICD-10-D-D23122, ICD-10-D-D2320, ICD-10-D-D2321, ICD-10-D-D2322, ICD-10-D-D2330, ICD-10-D-D2339, ICD-10-D-D234, ICD-10-D-D235, ICD-10-D-D2360, ICD-10-D-D2361, ICD-10-D-D2362, ICD-10-D-D2370, ICD-10-D-D2371, ICD-10-D-D2372, ICD-10-D-D239, ICD-10-D-D241, ICD-10-D-D242, ICD-10-D-D249, ICD-10-D-D250, ICD-10-D-D251, ICD-10-D-D252, ICD-10-D-D259, ICD-10-D-D260, ICD-10-D-D261, ICD-10-D-D267, ICD-10-D-D269, ICD-10-D-D270, ICD-10-D-D271, ICD-10-D-D279, ICD-10-D-D280, ICD-10-D-D281, ICD-10-D-D282, ICD-10-D-D287, ICD-10-D-D290, ICD-10-D-D291, ICD-10-D-D2921, ICD-10-D-D294, ICD-10-D-D3000, ICD-10-D-D3001, ICD-10-D-D3002, ICD-10-D-D3010, ICD-10-D-D303, ICD-10-D-D308, ICD-10-D-D309, ICD-10-D-D3100, ICD-10-D-D3101, ICD-10-D-D3102, ICD-10-D-D3112, ICD-10-D-D3121, ICD-10-D-D3122, ICD-10-D-D3130, ICD-10-D-D3131, ICD-10-D-D3132, ICD-10-D-D3140, ICD-10-D-D3141, ICD-10-D-D3142, ICD-10-D-D3160, ICD-10-D-D3161, ICD-10-D-D3190, ICD-10-D-D3191, ICD-10-D-D3192, ICD-10-D-D320, ICD-10-D-D321, ICD-10-D-D329, ICD-10-D-D330, ICD-10-D-D331, ICD-10-D-D332, ICD-10-D-D333, ICD-10-D-D334, ICD-10-D-D337, ICD-10-D-D339, ICD-10-D-D34, ICD-10-D-D3500, ICD-10-D-D3501, ICD-10-D-D3502, ICD-10-D-D351, ICD-10-D-D352, ICD-10-D-D360, ICD-10-D-D3610, ICD-10-D-D3612, ICD-10-D-D3613, ICD-10-D-D3614, ICD-10-D-D3615, ICD-10-D-D3616, ICD-10-D-D3617, ICD-10-D-D367, ICD-10-D-D369, ICD-10-D-D3701, ICD-10-D-D3702, ICD-10-D-D37030, ICD-10-D-D37032, ICD-10-D-D3705, ICD-10-D-D3709, ICD-10-D-D371, ICD-10-D-D372, ICD-10-D-D373, ICD-10-D-D374, ICD-10-D-D375, ICD-10-D-D376, ICD-10-D-D378, ICD-10-D-D379, ICD-10-D-D380, ICD-10-D-D381, ICD-10-D-D382, ICD-10-D-D383, ICD-10-D-D386, ICD-10-D-D390, ICD-10-D-D3910, ICD-10-D-D3911, ICD-10-D-D3912, ICD-10-D-D398, ICD-10-D-D399, ICD-10-D-D3A00, ICD-10-D-D3A012, ICD-10-D-D3A019, ICD-10-D-D3A020, ICD-10-D-D3A021, ICD-10-D-D3A026, ICD-10-D-D3A090, ICD-10-D-D3A092, ICD-10-D-D3A093, ICD-10-D-D3A098, ICD-10-D-D3A8, ICD-10-D-D400, ICD-10-D-D4010, ICD-10-D-D4011, ICD-10-D-D408, ICD-10-D-D409, ICD-10-D-D4100, ICD-10-D-D4101, ICD-10-D-D4102, ICD-10-D-D4110, ICD-10-D-D4111, ICD-10-D-D4112, ICD-10-

Complication	ICD-10-D Codes
	D-D4120, ICD-10-D-D4122, ICD-10-D-D413, ICD-10-D-D414, ICD-10-D-D419, ICD-10-D-D420, ICD-10-D-D429, ICD-10-D-D430, ICD-10-D-D432, ICD-10-D-D433, ICD-10-D-D434, ICD-10-D-D439, ICD-10-D-D440, ICD-10-D-D4410, ICD-10-D-D4411, ICD-10-D-D4412, ICD-10-D-D442, ICD-10-D-D443, ICD-10-D-D444, ICD-10-D-D447, ICD-10-D-D449
Hepatocellular adenoma	ICD-10-D-D134
Malignant neoplasm	ICD-10-D-C001, ICD-10-D-C004, ICD-10-D-C01, ICD-10-D-C020, ICD-10-D-C021, ICD-10-D-C022, ICD-10-D-C023, ICD-10-D-C024, ICD-10-D-C028, ICD-10-D-C029, ICD-10-D-C031, ICD-10-D-C039, ICD-10-D-C040, ICD-10-D-C048, ICD-10-D-C049, ICD-10-D-C050, ICD-10-D-C051, ICD-10-D-C052, ICD-10-D-C059, ICD-10-D-C060, ICD-10-D-C069, ICD-10-D-C07, ICD-10-D-C080, ICD-10-D-C089, ICD-10-D-C090, ICD-10-D-C091, ICD-10-D-C098, ICD-10-D-C099, ICD-10-D-C100, ICD-10-D-C101, ICD-10-D-C102, ICD-10-D-C108, ICD-10-D-C109, ICD-10-D-C111, ICD-10-D-C112, ICD-10-D-C113, ICD-10-D-C118, ICD-10-D-C119, ICD-10-D-C12, ICD-10-D-C130, ICD-10-D-C131, ICD-10-D-C132, ICD-10-D-C138, ICD-10-D-C139, ICD-10-D-C140, ICD-10-D-C148, ICD-10-D-C153, ICD-10-D-C154, ICD-10-D-C155, ICD-10-D-C158, ICD-10-D-C159, ICD-10-D-C160, ICD-10-D-C161, ICD-10-D-C162, ICD-10-D-C163, ICD-10-D-C165, ICD-10-D-C166, ICD-10-D-C168, ICD-10-D-C169, ICD-10-D-C170, ICD-10-D-C171, ICD-10-D-C172, ICD-10-D-C178, ICD-10-D-C179, ICD-10-D-C180, ICD-10-D-C181, ICD-10-D-C182, ICD-10-D-C183, ICD-10-D-C184, ICD-10-D-C185, ICD-10-D-C186, ICD-10-D-C187, ICD-10-D-C188, ICD-10-D-C189, ICD-10-D-C19, ICD-10-D-C20, ICD-10-D-C210, ICD-10-D-C211, ICD-10-D-C218, ICD-10-D-C220, ICD-10-D-C221, ICD-10-D-C222, ICD-10-D-C223, ICD-10-D-C227, ICD-10-D-C228, ICD-10-D-C229, ICD-10-D-C23, ICD-10-D-C240, ICD-10-D-C241, ICD-10-D-C248, ICD-10-D-C249, ICD-10-D-C250, ICD-10-D-C251, ICD-10-D-C252, ICD-10-D-C253, ICD-10-D-C254, ICD-10-D-C257, ICD-10-D-C258, ICD-10-D-C259, ICD-10-D-C260, ICD-10-D-C269, ICD-10-D-C300, ICD-10-D-C310, ICD-10-D-C311, ICD-10-D-C313, ICD-10-D-C318, ICD-10-D-C319, ICD-10-D-C320, ICD-10-D-C321, ICD-10-D-C322, ICD-10-D-C323, ICD-10-D-C328, ICD-10-D-C329, ICD-10-D-C33, ICD-10-D-C3400, ICD-10-D-C3401, ICD-10-D-C3402, ICD-10-D-C3410, ICD-10-D-C3411, ICD-10-D-C3412, ICD-10-D-C342, ICD-10-D-C3430, ICD-10-D-C3431, ICD-10-D-C3432, ICD-10-D-C3480, ICD-10-D-C3481, ICD-10-D-C3482, ICD-10-D-C3490, ICD-10-D-C3491, ICD-10-D-C3492, ICD-10-D-C37, ICD-10-D-C381, ICD-10-D-C382, ICD-10-D-C383, ICD-10-D-C384, ICD-10-D-C388, ICD-10-D-C390, ICD-10-D-C399, ICD-10-D-C4000, ICD-10-D-C4001, ICD-10-D-C4002, ICD-10-D-C4020, ICD-10-D-C4021, ICD-10-D-C4022, ICD-10-D-C4032, ICD-10-D-C4080, ICD-10-D-C4090, ICD-10-D-C410, ICD-10-D-C411, ICD-10-D-C412, ICD-10-D-C413, ICD-10-D-C414, ICD-10-D-C419, ICD-10-D-C4311, ICD-10-D-C4312, ICD-10-D-C4330, ICD-10-D-C4339, ICD-10-D-C434, ICD-10-D-C4351, ICD-10-D-C4359, ICD-10-D-C4360, ICD-10-D-C4361, ICD-10-D-C4362, ICD-10-D-C4370, ICD-10-D-C4371, ICD-10-D-C4372, ICD-10-D-C438, ICD-10-D-C439, ICD-10-D-C4400, ICD-10-D-C4401, ICD-10-D-C4402, ICD-10-D-C44101, ICD-10-D-C441092, ICD-10-D-C44111, ICD-10-D-C44112, ICD-10-D-C44119, ICD-10-D-C441191, ICD-10-D-C441192, ICD-10-D-C44122, ICD-10-D-C44129, ICD-10-D-C44209,

Complication	ICD-10-D Codes
	ICD-10-D-C44211, ICD-10-D-C44212, ICD-10-D-C44219, ICD-10-D-C44222, ICD-10-D-C44229, ICD-10-D-C44299, ICD-10-D-C44300, ICD-10-D-C44301, ICD-10-D-C44309, ICD-10-D-C44310, ICD-10-D-C44311, ICD-10-D-C44319, ICD-10-D-C44320, ICD-10-D-C44321, ICD-10-D-C44329, ICD-10-D-C4440, ICD-10-D-C4441, ICD-10-D-C4442, ICD-10-D-C44500, ICD-10-D-C44501, ICD-10-D-C44509, ICD-10-D-C44511, ICD-10-D-C44519, ICD-10-D-C44520, ICD-10-D-C44521, ICD-10-D-C44529, ICD-10-D-C44599, ICD-10-D-C44609, ICD-10-D-C44611, ICD-10-D-C44612, ICD-10-D-C44619, ICD-10-D-C44621, ICD-10-D-C44622, ICD-10-D-C44629, ICD-10-D-C44702, ICD-10-D-C44709, ICD-10-D-C44711, ICD-10-D-C44712, ICD-10-D-C44719, ICD-10-D-C44721, ICD-10-D-C44722, ICD-10-D-C44729, ICD-10-D-C44792, ICD-10-D-C44799, ICD-10-D-C4481, ICD-10-D-C4489, ICD-10-D-C4490, ICD-10-D-C4491, ICD-10-D-C4492, ICD-10-D-C4499, ICD-10-D-C450, ICD-10-D-C451, ICD-10-D-C452, ICD-10-D-C457, ICD-10-D-C459, ICD-10-D-C460, ICD-10-D-C461, ICD-10-D-C462, ICD-10-D-C463, ICD-10-D-C464, ICD-10-D-C4650, ICD-10-D-C4651, ICD-10-D-C4652, ICD-10-D-C467, ICD-10-D-C469, ICD-10-D-C470, ICD-10-D-C480, ICD-10-D-C481, ICD-10-D-C482, ICD-10-D-C488, ICD-10-D-C490, ICD-10-D-C4911, ICD-10-D-C4912, ICD-10-D-C4920, ICD-10-D-C4921, ICD-10-D-C4922, ICD-10-D-C493, ICD-10-D-C494, ICD-10-D-C495, ICD-10-D-C496, ICD-10-D-C498, ICD-10-D-C499, ICD-10-D-C49A0, ICD-10-D-C49A2, ICD-10-D-C49A3, ICD-10-D-C49A4, ICD-10-D-C49A9, ICD-10-D-C50011, ICD-10-D-C50012, ICD-10-D-C50019, ICD-10-D-C50021, ICD-10-D-C50029, ICD-10-D-C50111, ICD-10-D-C50112, ICD-10-D-C50119, ICD-10-D-C50121, ICD-10-D-C50211, ICD-10-D-C50212, ICD-10-D-C50219, ICD-10-D-C50222, ICD-10-D-C50311, ICD-10-D-C50312, ICD-10-D-C50319, ICD-10-D-C50411, ICD-10-D-C50412, ICD-10-D-C50419, ICD-10-D-C50511, ICD-10-D-C50512, ICD-10-D-C50519, ICD-10-D-C50611, ICD-10-D-C50612, ICD-10-D-C50619, ICD-10-D-C50811, ICD-10-D-C50812, ICD-10-D-C50819, ICD-10-D-C50829, ICD-10-D-C50911, ICD-10-D-C50912, ICD-10-D-C50919, ICD-10-D-C50921, ICD-10-D-C50922, ICD-10-D-C50929, ICD-10-D-C510, ICD-10-D-C511, ICD-10-D-C518, ICD-10-D-C519, ICD-10-D-C52, ICD-10-D-C530, ICD-10-D-C531, ICD-10-D-C538, ICD-10-D-C539, ICD-10-D-C541, ICD-10-D-C542, ICD-10-D-C543, ICD-10-D-C548, ICD-10-D-C549, ICD-10-D-C55, ICD-10-D-C561, ICD-10-D-C562, ICD-10-D-C569, ICD-10-D-C5700, ICD-10-D-C5701, ICD-10-D-C5702, ICD-10-D-C5710, ICD-10-D-C574, ICD-10-D-C577, ICD-10-D-C579, ICD-10-D-C600, ICD-10-D-C601, ICD-10-D-C602, ICD-10-D-C608, ICD-10-D-C609, ICD-10-D-C61, ICD-10-D-C6202, ICD-10-D-C6210, ICD-10-D-C6211, ICD-10-D-C6212, ICD-10-D-C6290, ICD-10-D-C6291, ICD-10-D-C6292, ICD-10-D-C6302, ICD-10-D-C6310, ICD-10-D-C638, ICD-10-D-C641, ICD-10-D-C642, ICD-10-D-C649, ICD-10-D-C651, ICD-10-D-C652, ICD-10-D-C659, ICD-10-D-C661, ICD-10-D-C662, ICD-10-D-C669, ICD-10-D-C670, ICD-10-D-C671, ICD-10-D-C672, ICD-10-D-C673, ICD-10-D-C674, ICD-10-D-C675, ICD-10-D-C677, ICD-10-D-C678, ICD-10-D-C679, ICD-10-D-C680, ICD-10-D-C688, ICD-10-D-C689, ICD-10-D-C6900, ICD-10-D-C6901, ICD-10-D-C6902, ICD-10-D-C6911, ICD-10-D-C6930, ICD-10-D-C6931, ICD-10-D-C6932, ICD-10-D-C6940, ICD-10-D-C6941, ICD-10-D-C6950, ICD-10-D-C6952, ICD-10-D-C6960, ICD-10-D-C6961, ICD-10-D-C6962, ICD-10-D-C6990, ICD-10-D-

Complication	ICD-10-D Codes
	C6991, ICD-10-D-C6992, ICD-10-D-C700, ICD-10-D-C701, ICD-10-D-C709, ICD-10-D-C710, ICD-10-D-C711, ICD-10-D-C712, ICD-10-D-C713, ICD-10-D-C714, ICD-10-D-C715, ICD-10-D-C716, ICD-10-D-C717, ICD-10-D-C718, ICD-10-D-C719, ICD-10-D-C720, ICD-10-D-C7230, ICD-10-D-C7231, ICD-10-D-C7232, ICD-10-D-C7259, ICD-10-D-C729, ICD-10-D-C73, ICD-10-D-C7400, ICD-10-D-C7401, ICD-10-D-C7402, ICD-10-D-C7412, ICD-10-D-C7490, ICD-10-D-C7491, ICD-10-D-C7492, ICD-10-D-C750, ICD-10-D-C751, ICD-10-D-C752, ICD-10-D-C753, ICD-10-D-C755, ICD-10-D-C758, ICD-10-D-C759, ICD-10-D-C760, ICD-10-D-C761, ICD-10-D-C762, ICD-10-D-C763, ICD-10-D-C7642, ICD-10-D-C7650, ICD-10-D-C7651, ICD-10-D-C7652, ICD-10-D-C768, ICD-10-D-C770, ICD-10-D-C771, ICD-10-D-C772, ICD-10-D-C773, ICD-10-D-C774, ICD-10-D-C775, ICD-10-D-C778, ICD-10-D-C779, ICD-10-D-C7800, ICD-10-D-C7801, ICD-10-D-C7802, ICD-10-D-C781, ICD-10-D-C782, ICD-10-D-C7830, ICD-10-D-C7839, ICD-10-D-C784, ICD-10-D-C785, ICD-10-D-C786, ICD-10-D-C787, ICD-10-D-C7880, ICD-10-D-C7889, ICD-10-D-C7900, ICD-10-D-C7901, ICD-10-D-C7902, ICD-10-D-C7910, ICD-10-D-C7911, ICD-10-D-C7919, ICD-10-D-C792, ICD-10-D-C7931, ICD-10-D-C7932, ICD-10-D-C7940, ICD-10-D-C7949, ICD-10-D-C7951, ICD-10-D-C7952, ICD-10-D-C7960, ICD-10-D-C7961, ICD-10-D-C7962, ICD-10-D-C7970, ICD-10-D-C7971, ICD-10-D-C7972, ICD-10-D-C7981, ICD-10-D-C7982, ICD-10-D-C7989, ICD-10-D-C799, ICD-10-D-C7A00, ICD-10-D-C7A010, ICD-10-D-C7A012, ICD-10-D-C7A019, ICD-10-D-C7A020, ICD-10-D-C7A021, ICD-10-D-C7A022, ICD-10-D-C7A025, ICD-10-D-C7A026, ICD-10-D-C7A029, ICD-10-D-C7A090, ICD-10-D-C7A091, ICD-10-D-C7A092, ICD-10-D-C7A093, ICD-10-D-C7A094, ICD-10-D-C7A095, ICD-10-D-C7A098, ICD-10-D-C7A1, ICD-10-D-C7A8, ICD-10-D-C7B00, ICD-10-D-C7B01, ICD-10-D-C7B02, ICD-10-D-C7B03, ICD-10-D-C7B04, ICD-10-D-C7B09, ICD-10-D-C7B8, ICD-10-D-C800, ICD-10-D-C801, ICD-10-D-C802, ICD-10-D-D0000, ICD-10-D-D0003, ICD-10-D-D0004, ICD-10-D-D0005, ICD-10-D-D0006, ICD-10-D-D0007, ICD-10-D-D0008, ICD-10-D-D002, ICD-10-D-D010, ICD-10-D-D011, ICD-10-D-D012, ICD-10-D-D013, ICD-10-D-D015, ICD-10-D-D017, ICD-10-D-D020, ICD-10-D-D0220, ICD-10-D-D0221, ICD-10-D-D0222, ICD-10-D-D0339, ICD-10-D-D034, ICD-10-D-D0352, ICD-10-D-D0359, ICD-10-D-D0361, ICD-10-D-D0362, ICD-10-D-D0371, ICD-10-D-D0372, ICD-10-D-D038, ICD-10-D-D039, ICD-10-D-D040, ICD-10-D-D0411, ICD-10-D-D0412, ICD-10-D-D0421, ICD-10-D-D0422, ICD-10-D-D0430, ICD-10-D-D0439, ICD-10-D-D044, ICD-10-D-D045, ICD-10-D-D0460, ICD-10-D-D0461, ICD-10-D-D0462, ICD-10-D-D0470, ICD-10-D-D0471, ICD-10-D-D0472, ICD-10-D-D048, ICD-10-D-D049, ICD-10-D-D0501, ICD-10-D-D0502, ICD-10-D-D0510, ICD-10-D-D0511, ICD-10-D-D0512, ICD-10-D-D0580, ICD-10-D-D0581, ICD-10-D-D0582, ICD-10-D-D0590, ICD-10-D-D0591, ICD-10-D-D0592, ICD-10-D-D060, ICD-10-D-D067, ICD-10-D-D069, ICD-10-D-D070, ICD-10-D-D071, ICD-10-D-D0730, ICD-10-D-D0739, ICD-10-D-D075, ICD-10-D-D090, ICD-10-D-D0910, ICD-10-D-D0919, ICD-10-D-D0922, ICD-10-D-D098, ICD-10-D-D099
Primary liver cancer	ICD-10-D-C220, ICD-10-D-C221, ICD-10-D-C222, ICD-10-D-C223, ICD-10-D-C224, ICD-10-D-C227, ICD-10-D-C228, ICD-10-D-C229
**Liver and/or kidney transplant**	

Complication	ICD-10-D Codes
Liver transplant	ICD-10-D-T8640, ICD-10-D-T8641, ICD-10-D-T8642, ICD-10-D-T8643, ICD-10-D-T8649, ICD-10-D-Z4823, ICD-10-D-Z944
Kidney transplant	ICD-10-D-T8610, ICD-10-D-T8611, ICD-10-D-T8612, ICD-10-D-T8613, ICD-10-D-T8619, ICD-10-D-Z4822, ICD-10-D-Z940

Abbreviations: GSDIa, glycogen storage disease type Ia; ICD-10-CM, *International Classification of Diseases, Tenth Revision–Clinical Modification.*

Table S2. Prevalence of Complications

	Pediatric		Adult		Total	
	**GSDIa (n=185)**	**Comparator (n=1850)**	**GSDIa (n=372)**	**Comparator (n=3720)**	**GSDIa (n=557)**	**Comparator (n=5570)**
**Any**	**167 (90.3)**	**815 (44.1)**	**356 (95.7)**	**2737 (73.6)**	**523 (93.9)**	**3552 (63.8)**
**Kidney and/or liver transplant**	**NR[a]**	**0**	**12 (3.2)**	**NR[a]**	**17 (3.1)**	**NR[a]**
Liver transplant	NR[a]	0	11 (3.0)	NR[a]	16 (2.9)	NR[a]
Kidney transplant	NR[a]	0	NR[a]	NR	NR[a]	NR[a]
**Hematologic**	**117 (63.2)**	**49 (2.7)**	**230 (61.8)**	**329 (8.8)**	**347 (62.3)**	**378 (6.8)**
Acidosis	37 (20.0)	NR[a]	64 (17.2)	24 (0.7)	101 (18.1)	27 (0.5)
Anemia due to enzyme disorders	81 (43.8)	0	151 (40.6)	NR[a]	232 (41.7)	NR[a]
Anemia	42 (22.7)	47 (2.5)	138 (37.1)	321 (8.6)	180 (32.3)	368 (6.6)
Iron deficiency anemia	22 (11.9)	16 (0.9)	62 (16.7)	127 (3.4)	84 (15.1)	143 (2.6)
**Hepatic**	**71 (38.4)**	**54 (2.9)**	**204 (54.8)**	**1074 (28.9)**	**275 (49.4)**	**1128 (20.3)**
Hepatomegaly	53 (28.7)	NR[a]	59 (15.9)	28 (0.8)	112 (20.1)	32 (0.6)
Hypoglycemia	50 (27.0)	22 (1.2)	42 (11.3)	32 (0.9)	92 (16.5)	54 (1.0)
Hyperlipidemia	20 (10.8)	28 (1.5)	168 (45.2)	1047 (28.2)	188 (33.8)	1075 (19.3)
**Renal**	**33 (17.8)**	**12 (0.7)**	**120 (32.3)**	**248 (6.7)**	**153 (27.5)**	**260 (4.7)**
Hyperuricemia	NR[a]	0	19 (5.1)	12 (0.3)	27 (4.9)	12 (0.2)
Dialysis	0	0	NR[a]	NR[a]	NR[a]	NR[a]
Kidney hypertrophy	NR[a]	0	NR[a]	0	NR[a]	0
Acute kidney failure	NR[a]	NR[a]	57 (15.3)	54 (1.5)	62 (11.1)	55 (1.0)

	Pediatric		Adult		Total	
	**GSDIa (n=185)**	**Comparator (n=1850)**	**GSDIa (n=372)**	**Comparator (n=3720)**	**GSDIa (n=557)**	**Comparator (n=5570)**
Proteinuria	NR[a]	NR[a]	21 (5.7)	31 (0.8)	28 (5.0)	38 (0.7)
Chronic kidney disease	NR[a]	NR[a]	48 (12.9)	88 (2.4)	55 (9.9)	90 (1.6)
Severe chronic kidney disease	NR[a]	0	16 (4.3)	19 (0.5)	17 (3.1)	19 (0.3)
Kidney stone	14 (7.6)	NR[a]	42 (11.3)	121 (3.3)	56 (10.1)	123 (2.2)
Focal segmental glomerulosclerosis	0	0	NR[a]	NR[a]	NR[a]	NR
**Nutritional**	**114 (61.6)**	**222 (12.0)**	**221 (59.4)**	**1152 (31.0)**	**335 (60.1)**	**1374 (24.7)**
Gastrostomy	55 (29.7)	NR[a]	12 (3.2)	NR[a]	67 (12.0)	NR[a]
Poor growth	41 (22.2)	70 (3.8)	NR[a]	NR[a]	48 (8.6)	75 (1.4)
Anorexia	NR[a]	NR[a]	18 (4.8)	15 (0.4)	26 (4.7)	25 (0.5)
Nutritional deficiency	59 (31.9)	44 (2.4)	150 (40.3)	594 (16.0)	209 (37.5)	638 (11.5)
Protein-calorie malnutrition	NR[a]	NR[a]	18 (4.8)	24 (0.7)	26 (4.7)	26 (0.5)
Iron deficiency	29 (15.7)	19 (1.0)	65 (17.5)	137 (3.7)	94 (16.9)	156 (2.8)
Vitamin D deficiency	33 (17.8)	20 (1.1)	90 (24.2)	458 (12.3)	123 (22.1)	478 (8.6)
Other nutritional deficiency	NR[a]	NR[a]	27 (7.3)	137 (3.7)	31 (5.6)	145 (2.6)
Obesity	35 (18.9)	114 (6.2)	116 (31.2)	718 (19.3)	151 (27.1)	832 (14.9)
**Digestive system**	**104 (56.2)**	**461 (24.9)**	**241 (64.8)**	**1173 (31.5)**	**345 (61.9)**	**1634 (29.3)**
Pancreatitis	NR[a]	0	11 (3.0)	11 (0.3)	12 (2.2)	11 (0.2)
Intestinal gas	NR[a]	20 (1.1)	38 (10.2)	95 (2.6)	48 (8.6)	115 (2.1)
Nausea and/or vomiting	81 (43.8)	235 (12.7)	133 (35.8)	340 (9.1)	214 (38.4)	575 (10.3)

	Pediatric		Adult		Total	
	GSDIa (n=185)	Comparator (n=1850)	GSDIa (n=372)	Comparator (n=3720)	GSDIa (n=557)	Comparator (n=5570)
Gallstones	NR[a]	0	29 (7.8)	72 (1.9)	32 (5.8)	72 (1.3)
Diarrhea	49 (26.5)	130 (7.0)	69 (18.6)	212 (5.7)	118 (21.2)	342 (6.1)
Abdominal pain	51 (27.6)	198 (10.7)	162 (43.6)	718 (19.3)	213 (38.2)	916 (16.5)
Gastroesophageal reflux disease	26 (14.1)	97 (5.2)	117 (31.5)	487 (13.1)	143 (25.7)	584 (10.5)
**Musculoskeletal**	**36 (19.5)**	**96 (5.2)**	**202 (54.3)**	**1036 (27.9)**	**238 (42.7)**	**1132 (20.3)**
Gout	NR[a]	0	44 (11.8)	81 (2.2)	45 (8.1)	81 (1.5)
Osteoporosis	NR[a]	NR[a]	27 (7.3)	76 (2.0)	32 (5.8)	77 (1.4)
Fatigue	30 (16.2)	92 (5.0)	130 (35.0)	726 (19.5)	160 (28.7)	818 (14.7)
Osteoarthritis	NR[a]	NR[a]	82 (22.0)	379 (10.2)	87 (15.6)	383 (6.9)
**Cardiovascular/cardiopulmonary**	**29 (15.7)**	**16 (0.9)**	**207 (55.7)**	**1045 (28.1)**	**236 (42.4)**	**1061 (19.1)**
Pulmonary hypertension	0	NR[a]	11 (3.0)	16 (0.4)	11 (2.0)	18 (0.3)
Hypertension	16 (8.7)	NR[a]	185 (49.7)	1009 (27.1)	201 (36.1)	1015 (18.2)
Atherosclerotic heart disease	0	NR[a]	32 (8.6)	157 (4.2)	32 (5.8)	158 (2.8)
**Neoplasm**	**18 (9.7)**	**104 (5.6)**	**153 (41.1)**	**842 (22.6)**	**171 (30.7)**	**946 (17.0)**
Malignant neoplasm	NR[a]	NR[a]	40 (10.8)	197 (5.3)	43 (7.7)	199 (3.6)
Primary liver cancer	0	0	NR[a]	0	NR[a]	0
Benign neoplasm	15 (8.1)	102 (5.5)	139 (37.4)	772 (20.8)	154 (27.7)	874 (15.7)
Hepatocellular adenoma	NR[a]	0	26 (7.0)	NR[a]	29 (5.2)	NR[a]
**Psychiatric/neurologic**	**24 (13.0)**	**176 (9.5)**	**143 (38.4)**	**1004 (27.0)**	**167 (30.0)**	**1180 (21.2)**

	Pediatric		Adult		Total	
	**GSDIa (n=185)**	**Comparator (n=1850)**	**GSDIa (n=372)**	**Comparator (n=3720)**	**GSDIa (n=557)**	**Comparator (n=5570)**
Seizure	NR[a]	NR[a]	NR[a]	15 (0.4)	NR[a]	18 (0.3)
Depression	12 (6.5)	74 (4.0)	89 (23.9)	540 (14.5)	101 (18.1)	614 (11.0)
Insomnia	NR[a]	22 (1.2)	37 (10.0)	223 (6.0)	39 (7.0)	245 (4.4)
Anxiety	18 (9.7)	139 (7.5)	98 (26.3)	715 (19.2)	116 (20.8)	854 (15.3)

Abbreviations: GSDIa, glycogen storage disease type Ia; NR, not reported.

[a]Patient counts of 1 to 10 are not reported for privacy reasons.

Table S3. Possible Complication Etiologies

Complication	Possible Etiology
**Digestive system**	
Abdominal pain	Large amounts of raw cornstarch have been found to cause abdominal pain; gallstones and pancreatitis may also contribute
Nausea and/or vomiting	Need for emergency care if oral feeds are not tolerated
Gastroesophageal reflux disease	Obesity and anxiety may contribute
Diarrhea	Large amounts of raw cornstarch can modify microbiota leading to diarrhea
Gastrostomy	Cornstarch and dietary management (continuous/nocturnal feeding)
Intestinal gas	Large amounts of raw cornstarch have been found to cause flatulence
Gallstones	Hyperlipidemia
Pancreatitis	Hyperlipidemia
**Nutritional**	
Nutritional deficiency	Dietary restrictions, management with cornstarch, digestive complications
Vitamin D deficiency	Dietary restriction of lactose and galactose
Iron deficiency	Cornstarch reduces iron bioavailability in the gastrointestinal tract
Protein-calorie malnutrition	Anorexia, liver and/or kidney disease, and cancer can result in disturbances of protein intake/metabolism
Other	Dietary restrictions, management with cornstarch, and digestive complications
Poor growth	Hypoglycemia, nutritional deficiency, and chronic acidosis
Obesity	Consumption of large amounts of raw cornstarch which is high in calories and reduced exercise capacity
Anorexia	Need for emergency care if unable to adhere to dietary management
**Hematologic**	
Anemia due to enzyme disorders	Possible miscoding or upcoding, as GSDIa is an enzyme disorder and anemia is a common complication
Anemia	Decreased production of erythropoietin due to renal insufficiency, chronic lactic acidosis, iron deficiency, and complications of hepatic adenoma
Iron deficiency anemia	Iron deficiency and bleeding due to altered platelet function
Acidosis	Progressive renal disease and lactic acidosis caused by repeated hypoglycemia
**Hepatic**	
Hepatomegaly	Accumulation of glucose in the liver
Hyperlipidemia	Increased lipid synthesis and decreased lipid serum clearance

Hypoglycemia	Deficiency of glucose-6-phosphatase results in impaired release of glucose from glycogen
**Cardiovascular/cardiopulmonary**	
Hypertension	Progressive renal disease
Atherosclerotic heart disease	Hypertension, hyperlipidemia, and obesity
Pulmonary hypertension	Abnormal production of serotonin, a vasoconstrictive amine (hypothesis)
**Musculoskeletal**	
Fatigue	Nutritional deficiency and hypoglycemia
Osteoarthritis	Obesity, gout, and reduced exercise capacity
Gout	Hyperuricemia stemming from altered renal tubular function
Osteoporosis	Lactic acidosis and cortisol release secondary to hypoglycemia and vitamin D deficiency and inadequate calcium intake due to dietary restrictions
**Psychiatric/neurologic**	
Anxiety	Diagnosis of rare disease and highest emotional burden in those with a rare disease where there are no available treatments
Depression	Diagnosis of rare disease and highest emotional burden in those with a rare disease where there are no available treatments
Insomnia	Ingestion of raw cornstarch every 4-5 hours including at night and recommended to prevent hypoglycemia in adults
Seizure	Hypoglycemia
**Renal**	
Acute kidney failure<br>Chronic kidney disease<br>Dialysis<br>Focal segmental glomerulosclerosis<br>Proteinuria	Activation of the renin–angiotensin system, prolonged oxidative stress, and profibrotic cytokines may be implicated in glomerular injury and progressive renal disease
Kidney hypertrophy	Accumulation of glycogen and fat in the kidneys
Kidney stone	Chronic acidosis, hypocitraturia, hypercalciuria, and hyperuricemia are thought to be contributing factors
Hyperuricemia	Hyperuricemia due to altered renal tubular function
**Neoplasm**	
Malignant neoplasm	
Primary liver cancer	Etiology of hepatocellular adenomas and transformation to hepatocellular carcinoma is multifactorial; insufficient metabolic control may play a role
Benign neoplasm	

Hepatocellular adenoma	Etiology of hepatocellular adenomas and transformation to hepatocellular carcinoma is multifactorial; insufficient metabolic control appears to play a role
**Liver and/or kidney transplant**	
Liver transplant	Accumulation of glycogen and fat in the liver, leading to progressive organ failure and tumors (adenomas and malignancies); may be preemptive to address underlying disease
Kidney transplant	Accumulation of glycogen and fat in the kidneys, leading to progressive organ failure

Figure S1. Complications in Children With GSDIa vs Comparators

	GSDIa, n (%)	Comparator, n (%)	OR (95% CI)
**Hematologic**	**117 (63.2)**	**49 (2.7)**	**63.2 (41.9–95.5)**
Acidosis	37 (20)	NR*	153.9 (46.9–505.0)
Anemia	42 (22.7)	47 (2.5)	11.3 (7.2–17.7)
Iron deficiency anemia	22 (11.9)	16 (0.9)	15.5 (8.0–30.0)
**Renal**	**33 (17.8)**	**12 (0.7)**	**33.3 (16.8–65.7)**
Kidney stone	14 (7.6)	NR*	75.7 (17.1–335.6)
**Cardiovascular/cardiopulmonary**	**29 (15.7)**	**16 (0.9)**	**21.3 (11.3–40.1)**
Hypertension	16 (8.7)	NR*	29.1 (11.2–75.3)
**Hepatic**	**71 (38.3)**	**54 (2.9)**	**20.7 (13.9–31.0)**
Hepatomegaly	53 (28.7)	NR*	185.3 (66.0–519.7)
Hypoglycemia	50 (27.0)	22 (1.2)	30.8 (18.1–52.3)
Hyperlipidemia	20 (10.8)	28 (1.5)	7.9 (4.4–14.3)
**Nutritional**	**114 (61.6)**	**222 (12.0)**	**11.8 (8.5–16.3)**
Gastrostomy	55 (29.7)	NR*	195.2 (69.7–546.9)
Poor growth	41 (21.2)	70 (3.8)	7.2 (4.8–11.0)
Nutritional deficiency	59 (31.9)	44 (2.4)	19.2 (12.5–29.6)
Iron deficiency	29 (15.7)	19 (1.0)	17.9 (9.8–32.7)
Vitamin D deficiency	33 (17.8)	20 (1.1)	19.9 (11.1–35.5)
Obesity	35 (18.9)	114 (6.2)	3.6 (2.4–5.4)
**Musculoskeletal**	**36 (19.5)**	**96 (5.2)**	**4.4 (2.9–6.7)**
Fatigue	30 (16.2)	92 (5.0)	3.7 (2.4–5.8)
**Digestive system**	**104 (56.2)**	**461 (24.9)**	**3.9 (2.8–5.3)**
Intestinal gas	NR*	20 (1.1)	5.2 (2.4–11.4)
Nausea and/or vomiting	81 (43.8)	235 (12.7)	5.4 (3.9–7.4)
Diarrhea	49 (26.5)	130 (7.0)	4.8 (3.3–6.9)
Abdominal pain	51 (27.6)	198 (10.7)	3.2 (2.2–4.5)
GERD	26 (14.1)	97 (5.2)	3.0 (1.9–4.7)
**Neoplasm**	**18 (9.7)**	**104 (5.6)**	**1.8 (1.1–3.1)**
Benign neoplasm	15 (8.1)	102 (5.5)	1.5 (0.9–2.7)
**Psychiatric/neurologic**	**24 (13.0)**	**176 (9.5)**	**1.4 (0.9–2.2)**
Depression	12 (6.5)	74 (4.0)	1.7 (0.9–3.1)
Insomnia	NR*	22 (1.2)	0.9 (0.2–3.9)
Anxiety	18 (9.7)	139 (7.5)	1.3 (0.8–2.2)

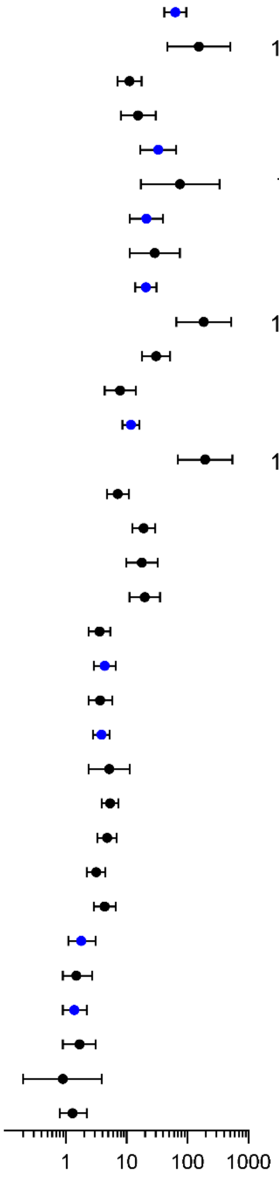

Abbreviations: GSDIa, glycogen storage disease type Ia; CI, confidence interval; NR, not reported; OR, odds ratio. *Patient counts of 1 to 10 were not reported for privacy reasons.

Figure S2. Complications in Adults With GSDIa vs Comparators

	GSDIa, n (%)	Comparator, n (%)	OR (95% CI)
**Liver and/or kidney transplant**	**12 (3.2)**	**NR***	**41.3 (11.6–147.0)**
Liver transplant	11 (3.0)	NR*	113.3 (14.6–880.1)
**Hematologic**	**230 (61.8)**	**329 (8.8)**	**16.7 (13.2–21.2)**
Acidosis	64 (17.2)	24 (0.7)	32.0 (19.7–51.9)
Anemia	138 (37.1)	321 (8.6)	6.3 (4.9–7.9)
Iron deficiency anemia	62 (16.7)	127 (3.4)	5.7 (4.1–7.8)
**Renal**	**120 (32.3)**	**248 (6.7)**	**6.7 (5.2–8.6)**
Hyperuricemia	19 (5.1)	12 (0.3)	16.6 (8.0–34.5)
Acute kidney failure	57 (15.3)	54 (1.5)	12.3 (8.3–18.1)
Proteinuria	21 (5.7)	31 (0.8)	7.1 (4.1–12.5)
Chronic kidney disease	48 (12.9)	88 (2.4)	6.1 (4.2–8.9)
Severe chronic kidney disease	16 (4.3)	19 (0.5)	8.8 (4.5–17.2)
Kidney stone	42 (11.3)	121 (3.3)	3.8 (2.6–5.5)
**Digestive system**	**241 (64.8)**	**1173 (31.5)**	**4.0 (3.2–5.0)**
Pancreatitis	11 (3.0)	11 (0.3)	10.3 (4.4–23.9)
Intestinal gas	38 (10.2)	95 (2.6)	4.3 (2.9–6.4)
Nausea and/or vomiting	133 (35.8)	340 (9.1)	5.5 (4.4–7.0)
Gallstones	29 (7.8)	72 (1.9)	4.3 (2.8–6.7)
Diarrhea	69 (18.6)	212 (5.7)	3.8 (2.8–5.1)
Abdominal pain	162 (43.6)	718 (19.3)	3.2 (2.6–4.0)
GERD	117 (31.5)	487 (13.1)	3.1 (2.4–3.9)
**Nutritional**	**221 (59.4)**	**1152 (31.0)**	**3.3 (2.6–4.1)**
Gastrostomy	12 (3.2)	NR*	124.0 (16.1–955.9)
Anorexia	18 (4.8)	15 (0.4)	12.6 (6.3–25.1)
Nutritional deficiency	150 (40.3)	594 (16.0)	3.6 (2.8–4.5)
Protein-calorie malnutrition	18 (4.8)	24 (0.7)	7.8 (4.2–14.6)
Iron deficiency	65 (17.5)	137 (3.7)	5.5 (4.0–7.6)
Vitamin D deficiency	90 (24.2)	458 (12.3)	2.3 (1.8–2.9)
Other nutritional deficiency	27 (7.3)	137 (3.7)	2.1 (1.3–3.1)
Obesity	116 (31.2)	718 (19.3)	1.9 (1.5–2.4)
**Cardiovascular/cardiopulmonary**	**207 (55.7)**	**1045 (28.1)**	**3.2 (2.6–4.0)**
Pulmonary hypertension	11 (3.0)	16 (0.4)	7.1 (3.3–15.3)
Hypertension	185 (49.7)	1009 (27.1)	2.7 (2.1–3.3)
Atherosclerotic heart disease	32 (8.6)	157 (4.2)	2.1 (1.4–3.2)
**Musculoskeletal**	**202 (54.3)**	**1036 (27.9)**	**3.1 (2.5–3.8)**
Gout	44 (11.8)	81 (2.2)	6.0 (4.1–8.9)
Osteoporosis	27 (7.3)	76 (2.0)	3.8 (2.4–5.9)
Fatigue	130 (35.0)	726 (19.5)	2.2 (1.8–2.8)
Osteoarthritis	82 (22.0)	379 (10.2)	2.5 (1.9–3.3)
**Hepatic**	**204 (54.8)**	**1074 (28.9)**	**3.0 (2.4–3.7)**
Hepatomegaly	59 (15.9)	28 (0.8)	24.9 (15.6–39.5)
Hypoglycemia	42 (11.3)	32 (0.9)	14.7 (9.1–23.6)
Hyperlipidemia	168 (45.2)	1047 (28.2)	2.1 (1.7–2.6)
**Neoplasm**	**153 (41.1)**	**842 (22.6)**	**2.4 (1.9–3.0)**
Malignant neoplasm	40 (10.8)	197 (5.3)	2.2 (1.5–3.1)
Benign neoplasm	139 (37.3)	772 (20.8)	2.3 (1.8–2.9)
Hepatocellular adenoma	26 (7.0)	NR*	279.5 (37.8–2065.6)
**Psychiatric/neurologic**	**143 (38.4)**	**1004 (27.0)**	**1.7 (1.4–2.1)**
Seizure	NR*	15 (0.4)	1.3 (0.3–5.9)
Depression	89 (23.9)	540 (14.5)	1.9 (1.4–2.4)
Insomnia	37 (10.0)	223 (6.0)	1.7 (1.2–2.5)
Anxiety	98 (26.3)	715 (19.2)	1.5 (1.2–1.9)

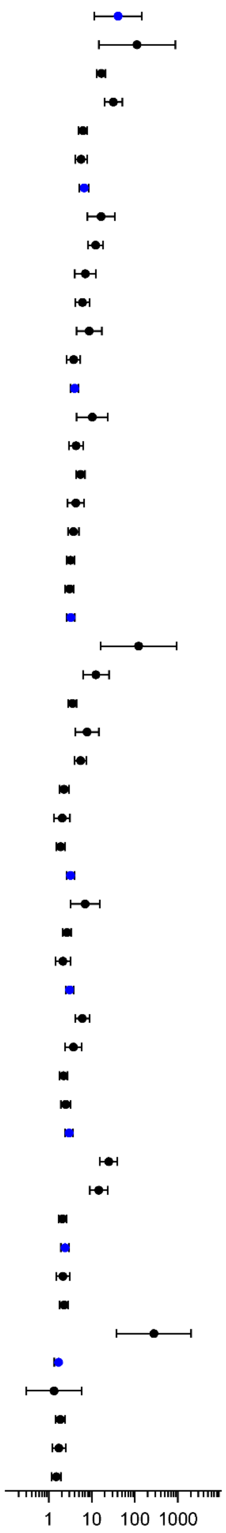

Abbreviations: CI, confidence interval; GSDIa, glycogen storage disease type Ia; NR, not reported; OR, odds ratio.

*Patient counts of 1-10 were not reported for privacy reasons.

Figure S3. Frequency of Complication Types by Patient Age in Patients With GSDIa (**A**) and in Comparators (**B**)

A

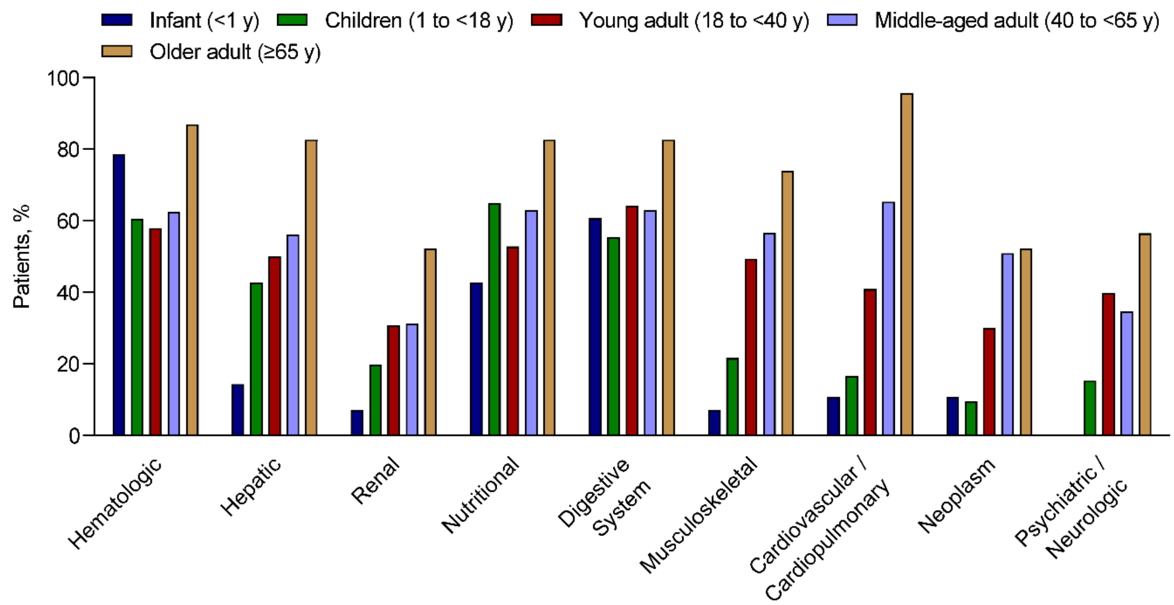

B

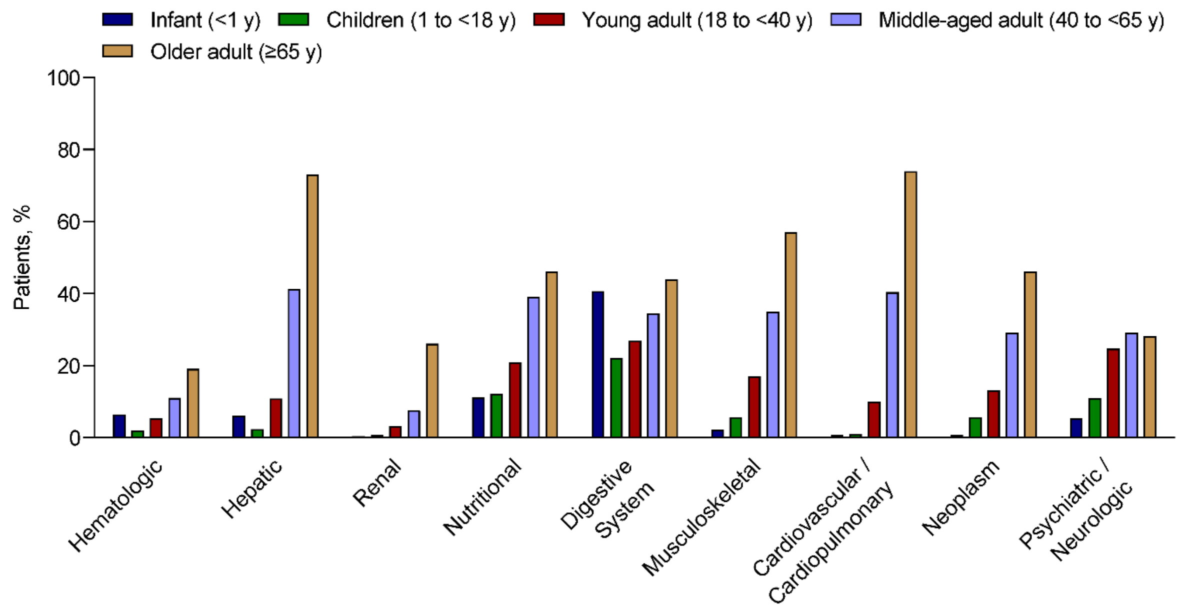

Abbreviation**:** GSDIa, glycogen storage disease type Ia.

Figure S4. Frequency of Hospitalizations by Patient Age in Patients With GSDIa (**A**) and in Comparators **(B)**

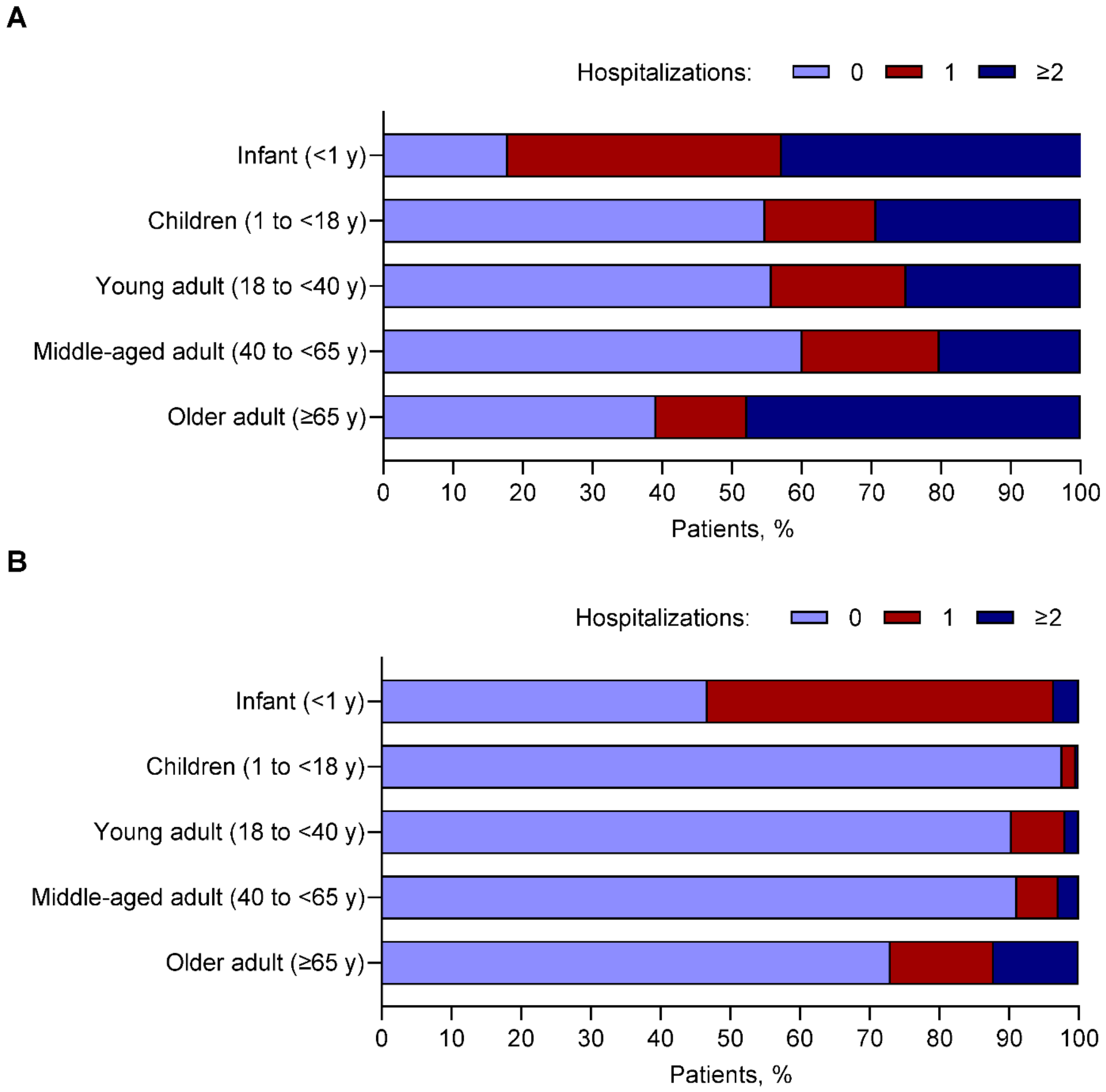

Abbreviation**:** GSDIa, glycogen storage disease type Ia.
